# Multifunctional Hydrogels with Broadband Electromagnetic Interference Shielding and Infrared Stealth Performance in Harsh Environments with Low Conductive Filler Content

**DOI:** 10.34133/research.1020

**Published:** 2026-02-06

**Authors:** Wenchong Ouyang, Lin Mei, Limin Xu, Chengwei Zhao, Yu Bai, Ziyang Zhao, Rongxin Tang, Tianzhi Luo, Zhengwei Wu

**Affiliations:** ^1^ School of Information Engineering, Nanchang University, Nanchang, Jiangxi 330031, China.; ^2^ Institute of Space Science and Technology, Nanchang University, Nanchang, Jiangxi 330031, China; ^3^ School of Nuclear Science and Technology, University of Science and Technology of China, Hefei 230026, China.; ^4^ CAS Key Laboratory of Mechanical Behavior and Design of Materials, Department of Modern Mechanics, CAS Center for Excellence in Complex System Mechanics, University of Science and Technology of China, Hefei 230026, China.; ^5^ Advanced Institute of Photonics Technology, School of Information Engineering, Guangdong University of Technology, Guangzhou 510006, China.; ^6^ Key Laboratory of Information and Structure Efficiency in Extreme Environment, the Ministry of Education of China, Xidian University, Xi’an 710071, China.; ^7^ Experimental Center of Engineering and Materials Sciences, University of Science and Technology of China, Hefei 230026, China.; ^8^ Joint Laboratory of Plasma Application Technology, Institute of Advanced Technology, University of Science and Technology of China, Hefei 230031, China.

## Abstract

Stretchable conductive hydrogel composites with infrared stealth and electromagnetic interference (EMI) shielding are in high demand for aerospace, military, and soft robotics. However, realizing stable and efficient performance with low conductive filler content under harsh conditions remains a substantial challenge. Herein, a flexible multifunctional double network hydrogels with high performance and environmental stability was constructed via transition metal carbide/nitride (MXene)/(NH_4_)_2_SO_4_-treated strategy-assisted ultrasonic dispersion and thermal polymerization method. The synergistic effect of MXene and (NH_4_)_2_SO_4_ within the hydrogel system led to a 3-fold enhancement in mechanical properties, 20-dB improvement in EMI shielding effectiveness, and 40% enhancement in the gauge factor with only 0.12 wt % conductive filler. Benefiting from its high conductivity, efficient thermal insulation, and composite network structure, the double network hydrogels maintain stable EMI shielding and infrared stealth performance under various harsh conditions including repeated stretching, prolonged water evaporation, low-temperature freezing, high-temperature heating, alcohol lamp flame exposure, and high-strain stretching. These findings demonstrate that the hydrogels combine ultra-low filler efficiency, environmental robustness, and multifunctional adaptability, making it a promising candidate for next-generation aerospace, military, and wearable electronic applications.

## Introduction

With the advancement of new-generation industrial technologies such as 5G/6G communications and the Internet of Things, electromagnetic interference (EMI) and radiation pollution have become increasingly severe [1]. This issue significantly impacts the global environment, equipment performance, information security, and human health [2]. The rise of intelligent equipment and the diversification of modern lifestyles have put forward new performance requirements on traditional EMI shielding materials such as sheets, films, and foams, including mechanical toughness, stretchability, and environmental stability, which are crucial for improving the effectiveness, reliability, and cost efficiency of EMI shielding materials [3,4]. Meanwhile, the rapid development of the aerospace and defense industries has introduced new technical requirements for broadband regulation of electromagnetic materials. Broadband electromagnetic control technologies spanning microwave, terahertz, and infrared bands can enhance the electromagnetic protection and infrared stealth performance, expanding their applicability in complex electromagnetic environments [5,6]. Therefore, there is an urgent demand to develop the multifunctional EMI shielding materials that simultaneously satisfy the demands of ultra-broadband EMI shielding, infrared stealth, high stretchability, excellent mechanical properties, good strain sensing, flame retardant, environmental stability, and cost efficiency.

Conductive hydrogels have attracted extensive attention as next-generation flexible EMI shielding materials due to their tissue-like mechanical properties, high conductivity, and multifunctional stretchability [7,8]. Their unique polymeric network with a high water content enables excellent deformation capacity and high-frequency EMI shielding performance, making them highly suitable for aerospace structural electronics, soft robotics, and stealth applications [9,10]. In recent years, nanomaterials such as graphene, liquid metal, MXene, and metal nanowires have been utilized as conductive fillers in combination with hydrogels that possess water-rich porous structures, thereby achieving flexible, stretchable, and excellent EMI shielding effects [11–14]. Increasing the content of conductive fillers markedly enhances the conductivity and EMI shielding effectiveness (SE) of hydrogels [15–20]. However, this improvement comes at the cost of mechanical properties such as strain, strength, and fracture energy, and high EMI SE relying on large filler loadings significantly raises material cost [19–22]. For instance, doubling the MXene content in MXene/polyvinyl alcohol (PVA) hydrogels can increase EMI SE by over 20 dB, while the strain and stress decreased significantly from 250% to 50% and from 0.14 MPa to 0.04 MPa, respectively [19]. A similar trend was observed in the Poly(potassium thioctate)/PVA/p-MXene, MXene/PAA (polyacrylic acid)–CS (chitosan), and CNC (cellulose nanocrystal)/PAA/liquid metal/MXene hydrogel systems, where increasing the conductive filler concentration greatly enhanced the EMI SE but inevitably led to a decline in strain, strength, and fracture energy [20–23]. Therefore, it is crucial to explore and design strategies to balance excellent mechanical properties and high EMI SE at low conductive content [24], a challenge that has yet to receive sufficient attention. In addition to achieving a desirable balance between mechanical sensing performance and high EMI shielding capability, another critical challenge is to realize broadband electromagnetic regulation. Current EMI SE shielding materials predominantly focus on the microwave band (X-Ka bands) [12–15,17–21,24–29], with limited research on the terahertz band [16,22,23], ultraviolet band [30], and infrared band [31–33]. In aerospace and defense applications, materials capable of broadband electromagnetic regulation across the microwave, terahertz, and infrared ranges are essential for achieving multispectral stealth and reliable signal protection.

Furthermore, the environmental stability of hydrogel-based EMI shielding materials remains underexplored. Hydrogels, with pure water as the primary dispersion medium, inevitably face environmental stability challenges such as water evaporation, freezing at temperatures below 0 °C, and thermal decomposition [34,35]. Additionally, the conductivity and thickness generally decrease during the stretching process [35,36], markedly reducing EMI SE and limiting practical longevity. Although the environmental stability of EMI shielding performance has been extensively studied in films and aerogels [37–40], it remains an area in the initial exploration stage for hydrogel shielding materials. Yu and colleagues [35,41] prepared MXene organic hydrogels and polypyrrole nanotube organic hydrogels using binary solvent systems such as glycerol/H_2_O and dimethyl sulfoxide (DMSO)/H_2_O, which showed excellent low-temperature resistance and anti-drying properties, maintaining EMI SE values far exceeding the 20-dB commercial standard after 7 d of evaporation and under freezing conditions. Lian and colleagues [15,24] reported that poly-acrylamide-acrylic acid and PEDOT:poly(3,4-ethylenedioxythiophene)/PVA hydrogels retained high EMI SE under strains ranging from 100% to 200%, attributed to the high tensile properties and high conductivity conferred by the hydrogels’ base formulation and conductive fillers, respectively. However, there is a notable lack of reports on hydrogel EMI shielding materials that maintain stable high EMI SE under multiple harsh conditions such as high stretchability, low temperatures, and dehydration. Such robustness is essential for expanding the application scope of composite hydrogel EMI shielding materials and enhancing their comprehensive performance. Therefore, achieving excellent EMI shielding performance in the microwave and terahertz ranges, as well as effective infrared shielding, under harsh environments and at low conductive filler contents remains another key challenge in the application of hydrogel EMI shielding materials.

Here, we propose a Ti₃C₂ MXene/(NH_4_)_2_SO_4_ treatment strategy to fabricate a multifunctional double network (DN) hydrogels composed of short CS chains and long polyelectrolyte (PE) chains synthesized from acrylic acid (AA) and 2-(dimethylamino) ethyl methacrylate (DMAEMA), which exhibits excellent flexibility, high stretchability, shape adaptability, mechanical sensing, and high EMI SE with environmental stability. Remarkably, the PE-CS hydrogels treated by MXene/(NH_4_)_2_SO_4_ (MNSPC) demonstrate significant improvements in both mechanical properties and EMI SE. By further optimizing the ratio of MXene/(NH_4_)_2_SO_4_ in the gel, MNSPC DN hydrogels achieve peak tensile strength, modulus, and strain of 1.24 MPa, 0.49 MPa, and 495%, respectively, at an ultra-low MXene content of 0.12 wt %, and exhibit exceptional ultra-wideband EMI shielding across X-band, Ku-band, Ka-band, and THz-band, as well as infrared shielding performance. Moreover, the MNSPC DN hydrogels still maintain stable EMI SE and infrared shielding performance, even under various harsh conditions such as 150% high tensile strain, long-term water evaporation, low-temperature freezing, and flame exposure. These findings provide valuable guidance for the development of low-cost, high-performance, environmentally stable, and stretchable EMI shielding hydrogels, demonstrating strong application potential in flexible electronics, bioelectronics, and smart sensors.

## Results

### Fabrication and characterization of MNSPC DN hydrogels

**Fig. 1.**
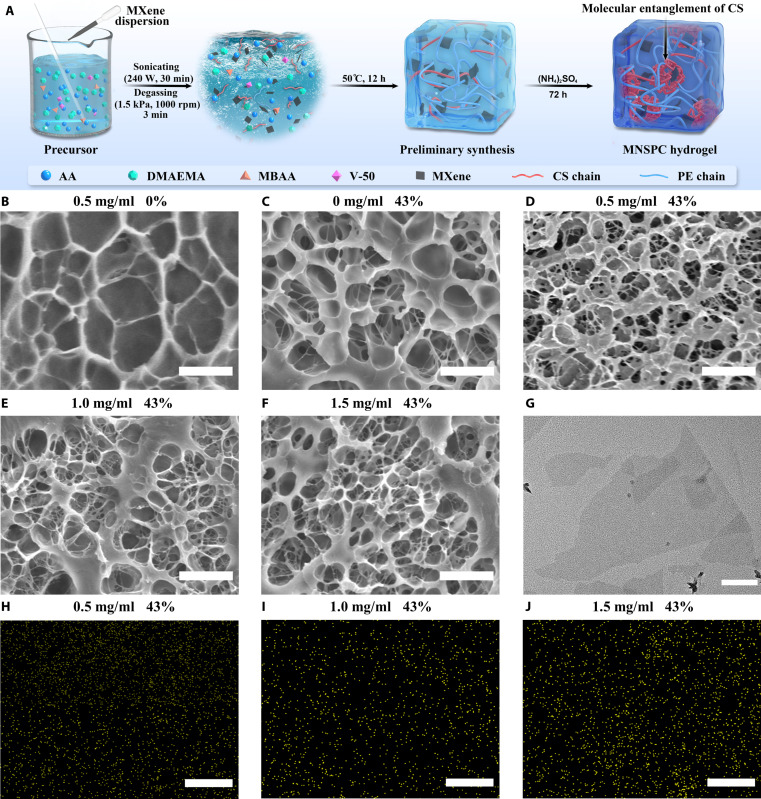
Fabrication and SEM, TEM, and EDS characterizations of MNSPC DN hydrogels. (A) Fabrication of MNSPC hydrogels. SEM images of hydrogels (B) before salting out (MPC hydrogels with 0.5 mg/ml MXene) and after salting out [MNSPC hydrogels with 43% (NH_4_)_2_SO_4_] under different MXene contents: (C) 0 mg/ml MXene [(NH_4_)_2_SO_4_/PE-CS (NSPC) hydrogels], (D) 0.5 mg/ml MXene, (E) 1.0 mg/ml MXene, and (F) 1.5 mg/ml MXene [(B) to (F): scale bar, 1 μm]. (G) TEM image of MXene nanosheets (scale bar, 200 nm). EDS mapping of titanium element distribution in MNSPC hydrogels after salting out with (H) 0.5 mg/ml MXene, (I) 1.0 mg/ml MXene, and (J) 1.5 mg/ml MXene [(H) to (J): scale bar, 10 μm].

MNSPC DN hydrogels were fabricated via ultrasonically assisted dispersion combined with thermal polymerization and salting-out methods, following the process illustrated in Fig. 1A. First, AA, CS, DMAEMA, V-50, Ti_3_C_2_ MXene suspension, and an appropriate volume of ultrapure water are mixed. The mixture is subjected to ultrasonically assisted dispersion to ensure uniform dispersion, particularly of the MXene, followed by vacuum mixing for degassing. PE networks are then formed using *N*,*N*-methylenebis(acrylamide) (MBAA) as a cross-linker, with polymerization initiated by V-50 at 50 °C for 12 h. Finally, the hydrogels undergo a salting-out process in a (NH_4_)_2_SO_4_ solution for 72 h, establishing a CS ionic network and triggering the Hofmeister effect, forming the MNSPC DN hydrogels. Briefly, the random copolymerization of AA and DMAEMA forms the PE chains, while (NH4)_2_SO_4_ induces the formation of the CS ionic network and triggers the Hofmeister effect, leading to molecular entanglement of CS chains. The interaction between MXene and the polymer network constitutes the entire hydrogel system.

Scanning electron microscopy (SEM), transmission electron microscopy (TEM), and energy-dispersive spectroscopy (EDS) characterization results reveal the porosity of hydrogels, formation of the CS ionic network, and uniform dispersion of MXene, respectively (Fig. 1). To minimize the effect of (NH_4_)_2_SO_4_ crystallization on the pores and microstructures of the hydrogels in SEM observations (Fig. S1), all samples containing (NH_4_)_2_SO_4_ are soaked in deionized water at 30 °C for 30 min before freeze-drying, ensuring more precise visualization of the network structures in the SEM images. SEM analysis of MXene/PE-CS (MPC) and MNSPC hydrogel samples (Fig. 1B and D) clearly demonstrates that the hydrogels exhibit a denser network and smaller pore sizes after salting out (Fig. S2), indicating the formation of the CS ionic network [42]. Similar trends are observed for MNSPC hydrogels with varying (NH_4_)_2_SO_4_ concentrations (Fig. S3), where an increase in (NH_4_)_2_SO_4_ concentration results in the contraction of the network pore size (Fig. S4). The influence of MXene content on the density of the hydrogel network and pore size after salting out is relatively minimal (Fig. 1C to F). Low concentrations of MXene lead to a slight reduction in pore size, while higher concentrations of MXene increase pore size (Fig. S5). The pore size of the hydrogels not only plays a crucial role in mechanical properties such as strength and toughness but also significantly affects the dispersion of MXene and its synergistic interactions [23,43]. TEM analysis reveals that the average length of MXene nanosheets is approximately 500 nm (Fig. 1G), which is smaller than the pore sizes of the hydrogels. This suggests that the pore structure provides a suitable environment for the efficient dispersion of MXene within the hydrogel matrix and the formation of conductive networks. EDS results further confirm that MXene can still be uniformly dispersed in the MNSPC DN hydrogels after high-concentration (NH_4_)_2_SO_4_ salting out (Fig. 1H to J and Fig. S6).

**Fig. 2.**
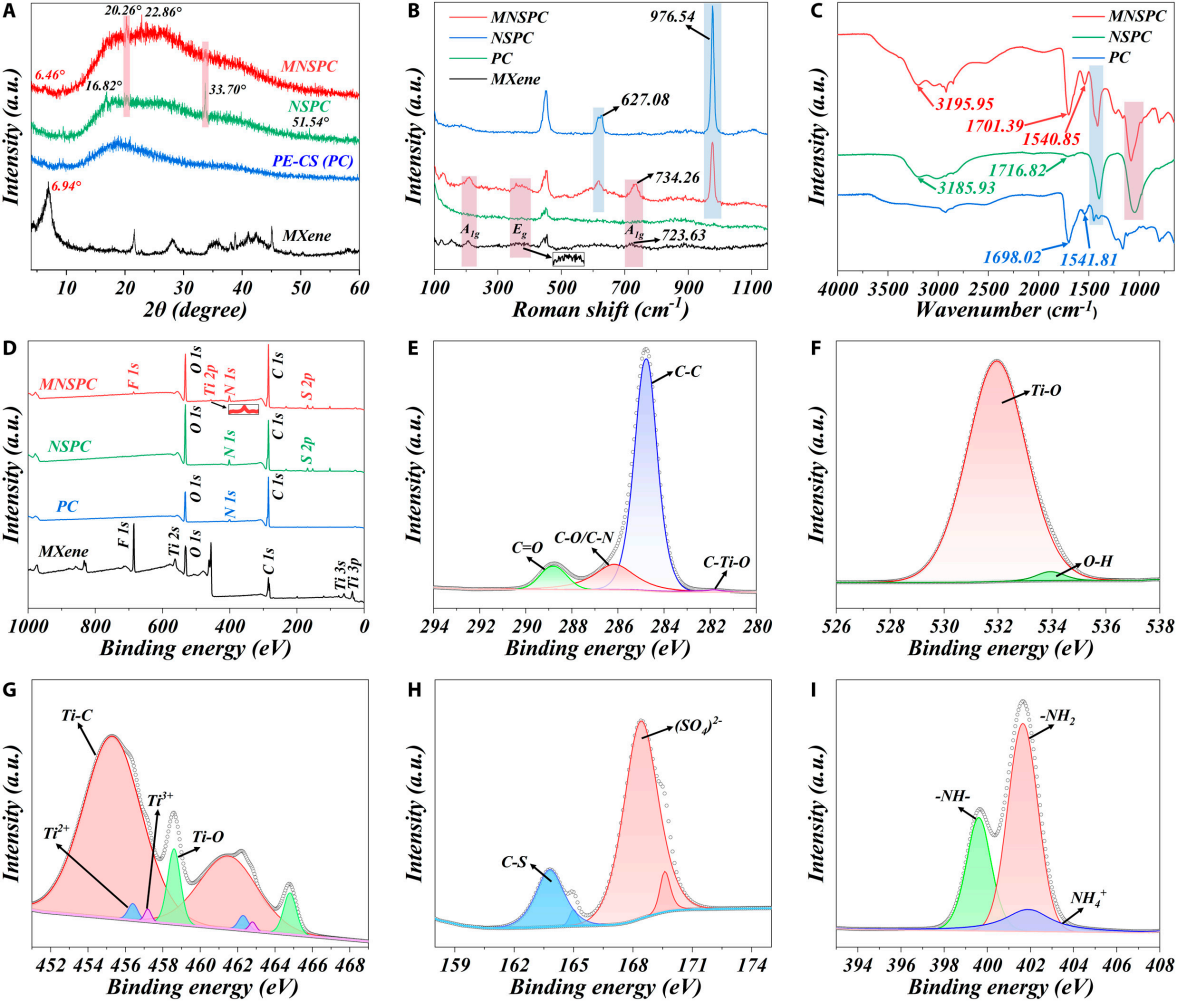
Comparison of characterization experimental results of MNSPC DN hydrogels and different reference samples. (A) XRD spectrum, (B) Raman spectra, (C) FTIR spectra, (D) XPS, and (E to I) high-resolution spectra of (E) C 1s, (F) O 1s, (G) S 2p, (H) Ti 2p, and (I) N 1s for MNSPC hydrogels.

X-ray diffraction (XRD), Fourier transform infrared (FTIR), Raman spectra, and x-ray photoelectron spectra (XPS) characterization experiments were performed to confirm the successful synthesis of MNSPC DN hydrogels, and to further explore the interactions between MXene, PE chains, CS chains, and (NH_4_)_2_SO_4_ within the hydrogel system. In the XRD characterization results, MNSPC, NSPC, and PC hydrogels exhibited similar peak profiles. The characteristic diffraction peak of MXene (002) in MNSPC shifted from 6.94° to 6.46°, indicating the successful integration of MXene, PE chains, and CS chains into the hydrogel structure (Fig. 2A) [16]. Compared to the PC hydrogel, the MNSPC (20.26°, 22.86°, 33.70°) and NSPC (16.82°, 20.26°, 33.70°, 51.54°) hydrogels displayed characteristic peaks consistent with (NH_4_)_2_SO_4_ (Fig. S7), demonstrating the successful incorporation of (NH_4_)_2_SO_4_ into the hydrogel structure. However, slight differences in the peak positions of the characteristic peaks shared with (NH_4_)_2_SO_4_ were observed in MNSPC and NSPC hydrogels, which could be attributed to the synergistic interactions between MXene, (NH_4_)_2_SO_4_, and the polymer network (PE and CS chains). In the Raman spectra, the MNSPC hydrogels exhibited characteristic peaks corresponding to MXene at approximately 210.13, 357.51, and 734.26 cm^−1^, associated with the A_1g_(Ti, C, O), E_g_, and A_1g_(C) modes (Fig. 2B). Compared to the PC hydrogels, both MNSPC and NSPC hydrogels exhibited several characteristic peaks of (NH_4_)_2_SO_4_ at 627.08 and 976.54 cm^−1^. Furthermore, in the MNSPC hydrogel, the A_1g_(C) peak of MXene shifted from 723.63 to 734.26 cm^−1^, suggesting potential interactions between MXene and the PE and CS chains [22,23]. FTIR analysis results revealed a shift in the −OH peak of the MNSPC hydrogels from 3,185.93 cm^−1^ in the NSPC hydrogels to 3,195.95 cm^−1^ (Fig. 2C), suggesting that the titanium-abundant surface groups of MXene facilitate the formation of additional hydrogen bonds [16,24]. The C=O peak in the NSPC hydrogels shifted from 1,698.02 cm^−1^ in the PC hydrogels to 1,716.82 cm^−1^, with a noticeable change in peak morphology, which can be attributed to the complex interactions between (NH_4_)_2_SO_4_ and the polymeric network. Compared to the NSPC hydrogels, the MNSPC hydrogels exhibited a significantly stronger and broader peak near 1,701.39 cm^−1^, which may be attributed to the C=O stretching vibrations of the oxygen-containing functional groups on the MXene surface [24]. Additionally, similar peak features to those of the NSPC and PC hydrogels at approximately 1,413.57 and 1,079.46 cm^−1^ were observed in the MNSPC hydrogels.

In the XPS spectra results, the coexistence of C 1s, N 1s, O 1s, S 2p, Ti 2p, and F 1s was observed in the MNSPC composite hydrogels. However, Ti 2p and F 1s were not detected in the NSPC hydrogels, and Ti 2p, F 1s, and S 2p were absent in the PC hydrogels, confirming the successful incorporation of MXene, PE chains, CS chains, and (NH_4_)_2_SO_4_ in the MNSPC hydrogel (Fig. 2D). The deconvolution of C 1s, O 1s, N 1s, S 2p, and Ti 2p peaks was performed to explore the interactions within the MNSPC DN hydrogel system. The C 1s spectrum showed peaks at 281.9, 284.8, 286.2, and 288.8 eV, corresponding to C–Ti–O, C–C, C–O/C–N, and C=O, respectively (Fig. 2E) [44]. The O 1s spectrum presented 2 main peaks at 531.9 and 534.0 eV, which can be attributed to Ti–O and O–H, respectively (Fig. 2F) [21,23]. Ti 2p spectrum exhibited 4 pairs of Ti 2p_3/2_ and Ti 2p_1/2_ doublets at 455.3/461.5 eV (Ti–C), 456.4/462.3 eV (Ti^2+^), 457.2/462.8 eV (Ti^3+^), and 458.6/464.8 eV (Ti–O) (Fig. 2G) [23,44]. S 2p spectrum showed 2 pairs of S 2p_3/2_ and S 2p_1/2_ doublets at 163.8/165.0 eV and 168.4/169.6 eV, corresponding to C–S and SO_4_^2−^, respectively (Fig. 2H) [22]. The existence of SO_4_^2−^ in the S 2p spectrum further confirms the successful incorporation of (NH_4_)_2_SO_4_ into MNSPC hydrogels. The N 1s XPS results showed that the peaks were located at 399.6, 401.65, and 401.86 eV, corresponding to -NH-, -NH_2_, and NH_4_^+^ in the MNSPC hydrogels (Fig. 2I) [22,44]. AA and DMAEMA were randomly copolymerized to form PE chains, and -COO^−^ in AA and -NH_3_^+^ in DMAEMA [45] were randomly distributed on the PE chains. -NH_2_ in CS will be protonated in an acidic environment to form -NH_3_^+^ [46], and these -NH_3_^+^ groups can also combine with -COO^−^ on the PE chain to form ionic bonds [47]. The ionic bonds established by Coulomb interactions between inter-chain and intra-chain groups with different charges, along with the molecular entanglement of CS chains induced by the Hofmeister effect from (NH_4_)_2_SO_4_ (Fig. 1B to F), significantly enhanced the mechanical properties of the hydrogel [22]. The above characterization results further confirmed the successful synthesis and interaction of PE, CS, MXene, and (NH_4_)_2_SO_4_ in the MNSPC DN hydrogels.

### Mechanical properties

**Fig. 3.**
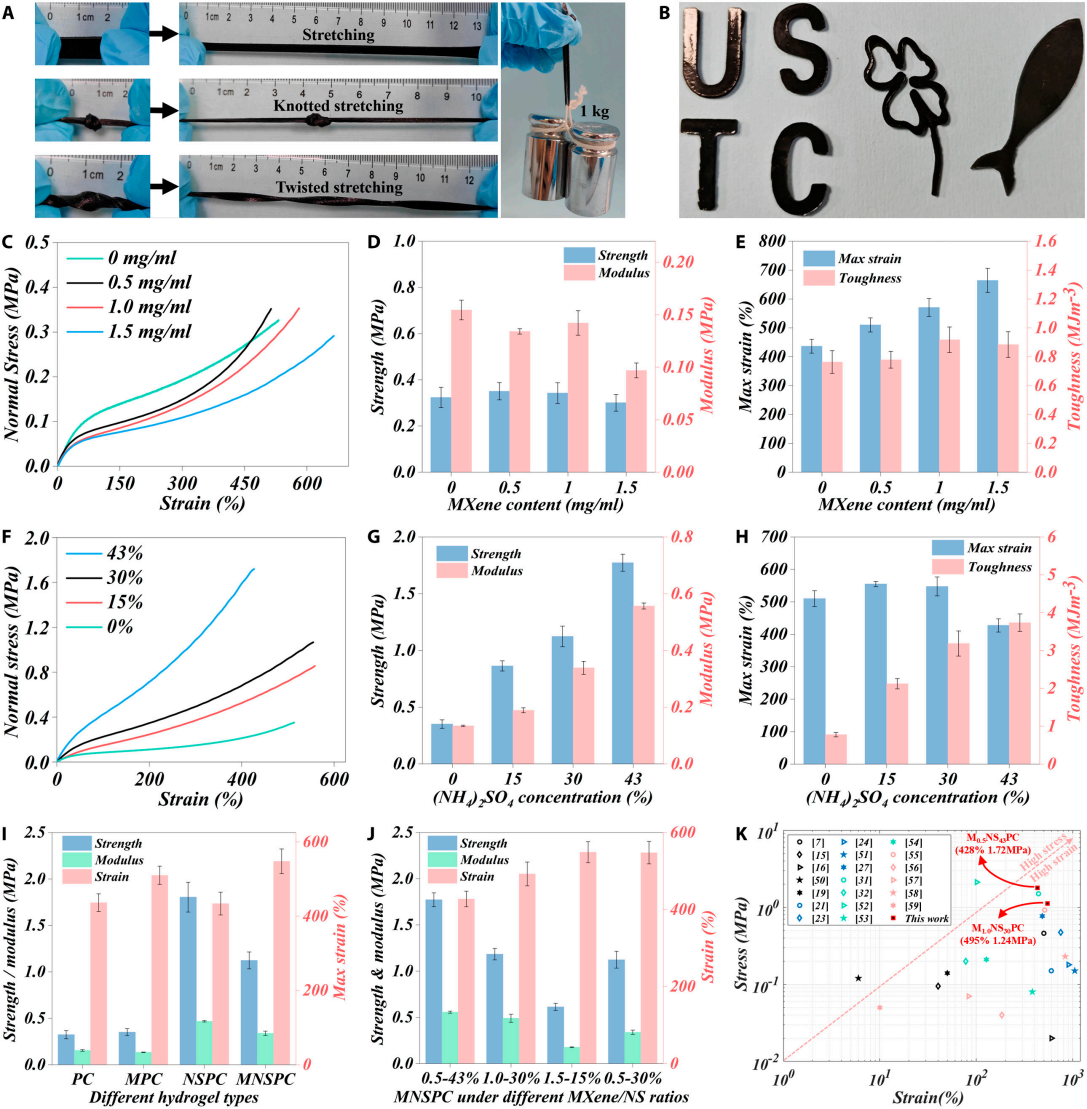
Mechanical properties of MNSPC DN hydrogels. (A) Stretching, knotting stretching, twisting stretching, and lifting a weight of 1 kg. (B) Shape adaptability, influence of MXene contents on the (C) stress–strain curves, (D) tensile strength and modulus, and (E) tensile strain and toughness of MPC hydrogels [(NH_4_)_2_SO_4_: 0]. Effect of (NH_4_)_2_SO_4_ contents on the (F) stress–strain curves, (G) tensile strength and modulus, and (H) tensile strain and toughness of MNSPC hydrogels (MXene: 0.5 mg/ml). Comparison of comprehensive mechanical properties of MNSPC hydrogels under (I) different hydrogel types and (J) different MXene/(NH_4_)_2_SO_4_ ratios. (K) Comparison of comprehensive mechanical properties between MNSPC hydrogels and representative EMI shielding hydrogels.

MNSPC hydrogels can withstand various deformations, including but not limited to stretching, knotting, and twisting, as well as lifting a 1-kg weight that is more than 1,000 times its own weight (Fig. 3A), while maintaining excellent shape adaptability (Fig. 3B). These phenomena revealed the superior mechanical properties of MNSPC DN hydrogels. The MXene/(NH_4_)_2_SO_4_ treatment strategy significantly enhances the mechanical properties of hydrogels, and the mechanical performance of the MNSPC DN hydrogels can be precisely modulated through the MXene/(NH₄)₂SO₄ ratio.

To explore the regulation pattern of MXene/(NH_4_)_2_SO_4_ concentrations on mechanical properties and the synergistic enhancement mechanism, the effects of MXene content, (NH_4_)_2_SO_4_ content, and the MXene/(NH_4_)_2_SO_4_ ratio on the tensile strength, modulus, strain, and toughness were systematically analyzed (Fig. 3C to J). A small MXene content (from 0 to 0.5 mg/ml) slightly increased the strength, modulus, maximum strain, and toughness of hydrogels. However, with the continuous rise of MXene content to 1.5 mg/ml, the strength of MPC hydrogels decreases from 0.35 to 0.30 MPa, the modulus drops from 0.15 to 0.10 MPa (Fig. 3C and D), and the toughness declines from 0.92 to 0.88 MJ/m^3^ (Fig. 3E). The maximum strain of MPC hydrogels increases monotonically from 436% to 664% with increasing MXene contents (Fig. 3C and E). Overall, the increase in MXene conductive filler tends to reduce the comprehensive mechanical properties of MPC hydrogels, and similar phenomena have also been observed in studies by other researchers [20–24].

The further introduction of (NH_4_)_2_SO_4_ into the MPC hydrogels (forming MNSPC hydrogels) significantly enhances their mechanical properties, with the strengthening effect far surpassing that of MXene. As the concentration of (NH_4_)_2_SO_4_ increases from 0% to 43%, the strength of MNSPC DN hydrogels increases from 0.35 to 1.77 MPa, the modulus from 0.13 to 0.56 MPa (Fig. 3F and G), and the toughness from 0.78 to 3.74 MJ/m^3^ (Fig. 3H). This remarkable improvement in mechanical performance is attributed to the formation of a DN and the strengthening of first network by the increase in the density of ionic bonds. The denser network structure and smaller pore sizes observed in the SEM images of the MPC hydrogels treated with (NH₄)₂SO₄ (MNSPC) provide additional support for this explanation (Fig. 1B to D). Additionally, the Hofmeister effect induced by (NH_4_)_2_SO_4_ enhances the molecular entanglement of CS chains, promoting the self-entanglement of the CS network and its interpenetration with the PE network [48,49]. Clearly, compared with PC and MPC hydrogels, the MXene/(NH_4_)_2_SO_4_-treated hydrogels (MNSPC) exhibited a 3-fold increase in strength, modulus, and toughness, along with about 120% slight improvement in maximum strain (Fig. 3I). Although the NSPC hydrogel also demonstrates a significant improvement in overall mechanical properties, its strain-stretching performance is notably inferior to that of the MNSPC hydrogels, which is critical for the development of stretchable hydrogel shielding materials.

Notably, the maximum strain of MNSPC increases from 510% to 548% as the concentration of (NH_4_)_2_SO_4_ rises from 0% to 30%, but significantly decreases to 427% when (NH_4_)_2_SO_4_ content reaches saturation at 43% (Fig. 3F and H). Excessive ions formed by excessive salting out lead to excessive cross-linking and inhomogeneity between polymer chains, thus forming a denser but less flexible network structure, which is reflected in the larger error bars observed in the mechanical performance results of high-concentration (NH_4_)_2_SO_4_ hydrogels (Fig. 3G and H and Fig. S8). Although this dense structure improves the rigidity of the hydrogel, it may also limit its elasticity and cause a decrease in strain capacity. On the other hand, the salting-out effect induced by excessive ammonium sulfate renders the hydrogel denser and less swellable, thereby limiting its stretchability. Additionally, the influence of MXene content observed in MNSPC hydrogels differed significantly from that in MPC hydrogels, suggesting a potential synergistic interaction between (NH_4_)_2_SO_4_ and MXene.

Therefore, a further investigation of the synergistic effects between MXene and (NH_4_)_2_SO_4_ content is essential for dynamically controlling the mechanical properties of MNSPC hydrogels and achieving optimal performance. The results of MNSPC hydrogels with varying MXene content at a high-concentration (NH₄)₂SO₄ of 43% indicated that the overall mechanical properties were minimally influenced by MXene, despite slight increases in strain and toughness as the increasing MXene content (Fig. S8A to C). However, this impact was nearly negligible relative to the excellent overall performance. Analysis of MNSPC hydrogels with 1.5 mg/ml MXene under different (NH_4_)_2_SO_4_ concentrations revealed a significant increase in strength, modulus, and toughness with the rise in (NH_4_)_2_SO_4_, accompanied by a consistent decrease in strain (Fig. S8D to F). Although the strength, modulus, and toughness reached their highest values at a concentration of 43% (NH_4_)_2_SO_4_, the more critical stretchability was reduced to 433%. A similar pattern was observed in hydrogels with lower MXene content (0.5 mg/ml), where strain initially increased with rising (NH₄)₂SO₄ contents but exhibited a marked decrease at the higher 43% concentration (Fig. 3F to H). Based on the trends observed under high (NH_4_)_2_SO_4_ concentrations (43%) with varying MXene content, as well as under different (NH_4_)_2_SO_4_ concentrations with both high (1.5 mg/ml) and low (0.5 mg/ml) MXene contents (Fig. 3 and Fig. S8), and considering strain stretchability as the primary performance criterion, M_0.5_NS_43_PC, M_1.0_NS_43_PC, M_1.5_NS_15_PC, and M_0.5_NS_30_PC were selected for further comparison to explore the optimal MXene/(NH_4_)_2_SO_4_ ratio. Notably, M_0.5_NS_30_PC hydrogels exhibited the highest stretchability at 548% with the lowest MXene content (0.5mg/ml), although its strength and modulus were slightly lower than M_0.5_NS_43_PC and M_1.0_NS_43_PC hydrogels, still achieving excellent values of 1.12 MPa (strength) and 0.34 MPa (modulus), respectively (Fig. 3J).

Overall, the strength, modulus, toughness, and strain of hydrogels treated with the MXene/(NH_4_)_2_SO_4_ strategy were significantly enhanced. The MNSPC hydrogels with an optimal ratio of 1.0 mg/ml MXene and 30% (NH_4_)_2_SO_4_ (M_1.0_NS_30_PC) achieved exceptional mechanical performance, with strain, strength, modulus, and toughness reaching 495%, 1.24 MPa, 0.49 MPa, and 3.19 MJm^−3^, surpassing most hydrogel shielding materials reported so far (Fig. 3K and Table S1) [7,15,16,19,21,23,24,27,31,32,50–59].

### Strain sensitivity sensor properties

**Fig. 4.**
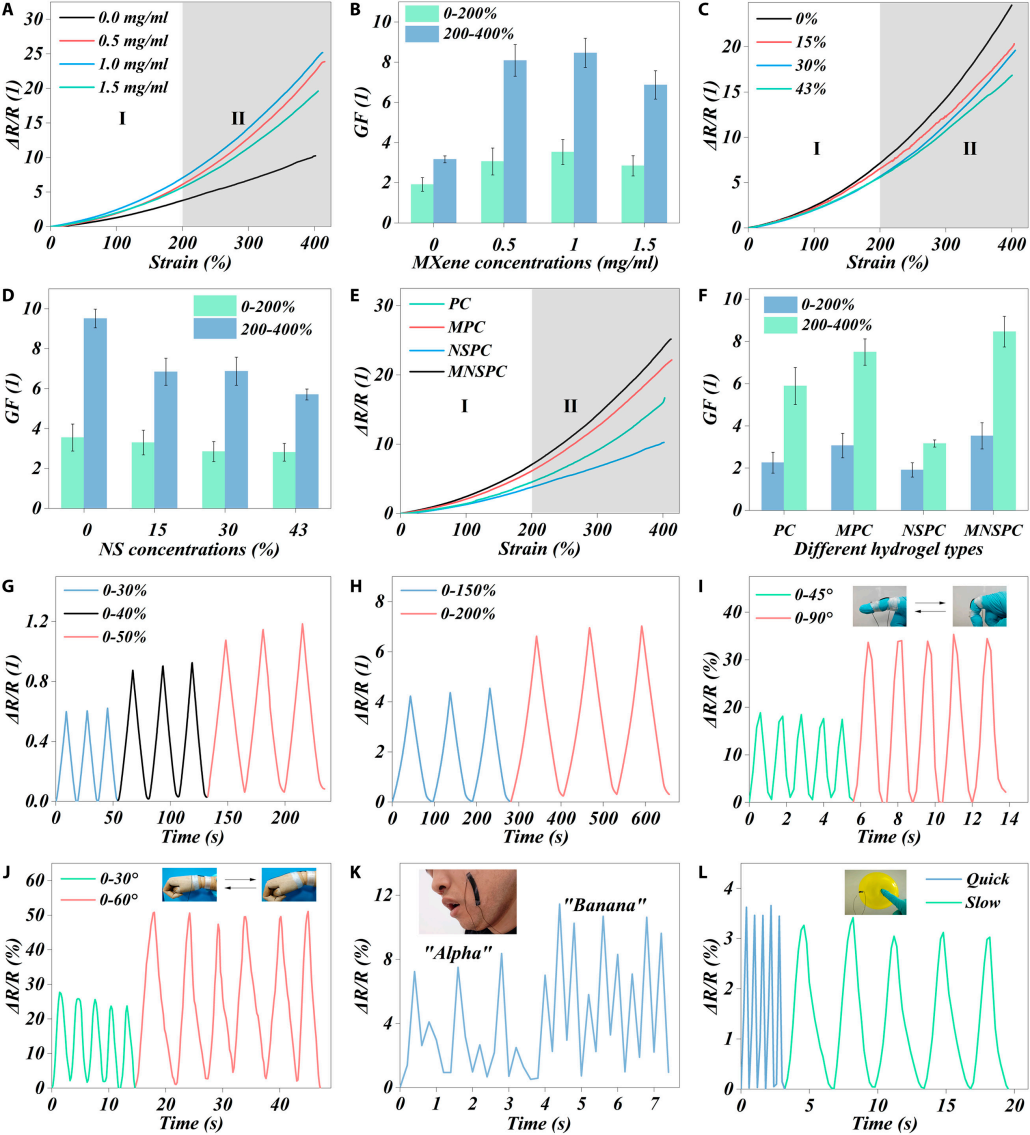
Strain sensitivity sensor properties and stability of MNSPC hydrogel under different component concentrations. (A) Relative resistance–strain curves and (B) GF in the range of 0% to 200% and 200% to 400% of MNSPC hydrogels with different MXene contents. (C) Relative resistance–strain curves and (D) GF in the range of 0% to 200% and 200% to 400% of MNSPC hydrogels with different (NH_4_)_2_SO_4_ contents. (E) Relative resistance–strain curves and (F) GF in the range of 0% to 200% and 200% to 400% of MNSPC hydrogels under different hydrogel types. Real-time Δ*R*/*R* curves recorded during cycles under (G) lower strain and (H) higher strain. Real-time Δ*R*/*R* curves of (I) finger grasping and (J) wrist rotation under different angles, (K) facial movement of speaking different words, and (L) balloon under pressure at different speeds.

The MNSPC hydrogel exhibited significant potential in deformation sensing applications due to its excellent conductivity (Fig. S9) [19–23]. The gauge factor (GF) was measured to assess the influence of MXene and (NH_4_)_2_SO_4_ on the strain sensor properties of the MNSPC hydrogel. The incorporation of MXene led to a marked increase in GF. However, only a slight decrease was observed with further increases in MXene concentration, with the maximum GF occurring at a concentration of 1 mg/ml (Fig. 4A and B). Conversely, the introduction of (NH_4_)_2_SO_4_ resulted in a significant reduction in GF, with a progressive decline as the (NH_4_)_2_SO_4_ concentration increased (Fig. 4C and D). Similar trends were observed when comparing the GF results across different hydrogel types. MPC hydrogels exhibited higher GF values than did PC hydrogels, while NSPC hydrogels showed lower GF values (Fig. 4E and F). However, the MNSPC hydrogel exhibited a distinctly higher GF than other hydrogels (Fig. 4E and F), attributed to the synergistic effect of MXene and ammonium sulfate, which optimally balanced both conductivity and mechanical properties (Fig. 3 and Fig. S9). Notably, in the higher strain range (200% to 400%), the maximum GF reached 8.47 ± 0.71, surpassing most reported EMI shielding materials (Table S2) [3,35,52,60–80].

In addition, the stability and repeatability of the sensor are critical for practical applications. The changes in resistance under different strain cycles demonstrated the excellent reproducibility and stability of the MNSPC hydrogel sensor (Fig. 4G and H). The practical feasibility of the MNSPC hydrogel sensor was further validated by integrating the hydrogel with a light-emitting diode (LED) and applying it to various body parts. The brightness of the LED significantly decreased as the hydrogel was stretched, owing to the increased resistance from the stretching, which reduced the current in the circuit (Fig. S10). The MNSPC hydrogel sensor effectively monitored finger bending and wrist rotation at different angles, with significant changes in the GF indicating its superior sensitivity (Fig. 4I and J). The sensor also detected facial movements, showing distinct resistance changes when words such as “Alpha” and “Banana” were spoken (Fig. 4K). Moreover, real-time resistance responses showed notable differences when a balloon was compressed at different speeds (Fig. 4L). The above results on stability, repeatability, and practical demonstrations confirm that the MNSPC hydrogel can be an excellent candidate for next-generation flexible strain sensors.

### Ultra-broadband EMI shielding performance and environmental stability

Conductive hydrogels are promising candidates for flexible EMI shielding materials due to their porous structure, hydrophilic environments, and high conductivity [19,20]. The MXene/(NH_4_)_2_SO_4_ treatment strategy alters the microstructure of MNSPC DN hydrogels and enhances their conductivity, which is crucial for improving EMI shielding performance [21–26].

**Fig. 5.**
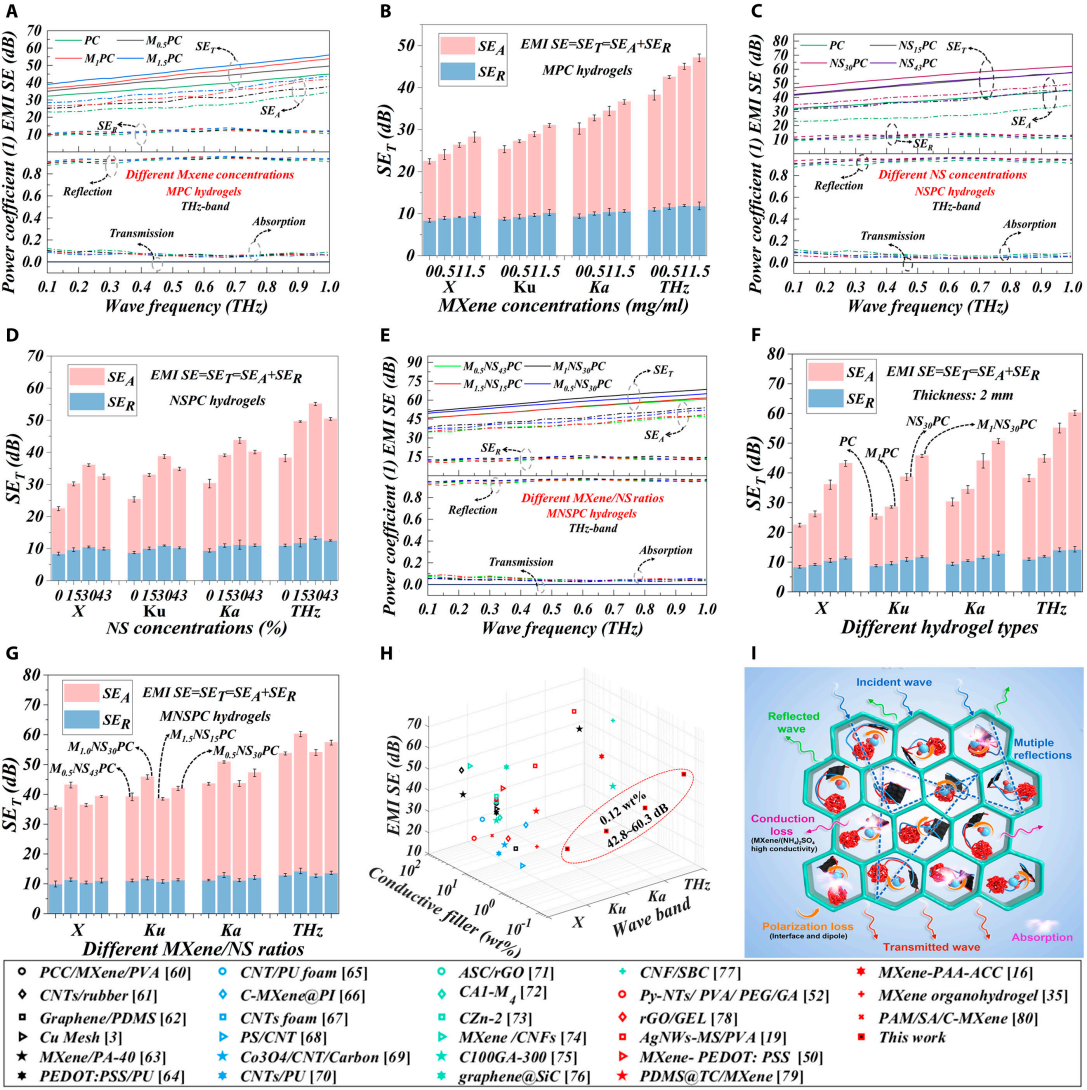
EMI shielding performance and related mechanism analysis of MNSPC DN hydrogels under different conditions. (A) EMI SE and power coefficient in THz-band across various MXene concentrations. (B) Influence analysis of MXene on EMI SE in different wave bands. (C) EMI SE and power coefficient in THz-band across various (NH_4_)_2_SO_4_ concentrations. (D) Influence analysis of (NH_4_)_2_SO_4_ concentrations on EMI SE in different wave bands. (E) EMI SE and power coefficient in THz-band under various MXene/(NH_4_)_2_SO_4_ ratios. Comparison of ${\mathrm{SE}}_{R},{\mathrm{SE}}_{A},\text{and}\;{\mathrm{SE}}_{T}$ in different wave bands under (F) different hydrogel types and (G) MXene/(NH_4_)_2_SO_4_ ratios. (H) Comparison of EMI SE under conductive fillers. (I) Analysis of EMI shielding mechanism.

Test results of the dynamic microwave and terahertz time-domain spectroscopy (THz-TDS) system reveal that the EMI SE of MPC hydrogels significantly increases across the X-band to the THz-band with the rising content of MXene (Fig. 5A and Fig. S11), and the enhancement effect of MXene in the THz-band is slightly higher than that in low-frequency bands such as the X-band (Fig. 5B). The enhancement in EMI SE is attributed to the increased conductivity induced by MXene (Fig. S9A), which effectively promotes the formation of a conductive network. The introduction of (NH_4_)_2_SO_4_ further elevates the EMI SE of NSPC hydrogels, significantly surpassing that of PC hydrogels. However, with the increase of (NH_4_)_2_SO_4_ concentrations, the EMI SE of NSPC hydrogels exhibits a nonmonotonic trend, initially increasing and then decreasing (Fig. 5C and Fig. S12). The maximum EMI SE enhancement, ranging from 13.6 to 16.9 dB across different frequency bands, is observed at a 30% (NH_4_)_2_SO_4_ concentrations (Fig. 5D). This behavior arises from the sharp conductivity increase induced by (NH_4_)_2_SO_4_ concentration, with the highest conductivity corresponding to the optimal EMI SE at this concentration (Fig. S9B). Notably, a substantially stronger influence is observed for (NH_4_)_2_SO_4_ content relative to MXene (Fig. 5B and D). This effect originates from its superior conductivity, ionic polarization effects, and excellent dispersion and uniformity of (NH_4_)_2_SO_4_ (Fig. S11A and B), which avoids issues such as layer stacking and local uniformity of MXene, especially in broadband applications.

The synergistic treatment of MNSPC hydrogels with MXene/(NH_4_)_2_SO_4_ produced an EMI SE enhancement exceeding 20 dB, markedly greater than that achieved by either MXene or (NH_4_)_2_SO_4_ component alone (Fig. 5E and F). Although both MXene and (NH_4_)_2_SO_4_ individually contribute to improved EMI shielding performance, excessive concentrations lead to a decrease in EMI SE. Additionally, the relative proportions of MXene and (NH₄)₂SO₄ significantly affect their synergistic effect. For instance, a significant decline in EMI SE is observed as (NH_4_)_2_SO_4_ concentration increases from 30% to saturation with 43% (Fig. 5C and D). Thus, optimizing the MXene/(NH_4_)_2_SO_4_ ratio is critical for achieving high EMI SE and mechanical performance with minimal conductive filler content. EMI SE and power coefficient under different MXene/(NH_4_)_2_SO_4_ ratios were further systematically analyzed, including high MXene with 1.5 mg/ml and low (NH_4_)_2_SO_4_ with 15% (M_1.5_NS_15_PC), low MXene with 0.5 mg/ml and high (NH_4_)_2_SO_4_ with 43% (M_0.5_NS_43_PC), and moderate MXene with 1.0 mg/ml and moderate (NH_4_)_2_SO_4_ with 30% (M_1.0_NS_30_PC). The M_1.0_NS_30_PC hydrogel exhibits higher SE_R_ and SE_A_ contributions than do M_1.5_NS_15_PC and M_0.5_NS_43_PC hydrogels, resulting in higher EMI SE (Fig. 5E and G and Fig. S13). This improvement can be attributed to the optimal balance between electronic and ionic conductivity, polarization effects, and conductive loss achieved with moderate concentrations of MXene and (NH_4_)_2_SO_4_, providing the best EMI shielding performance. Further increasing (NH_4_)_2_SO_4_ content leads to excessive ion concentration, causing the conductivity to decrease (Fig. S9B). On the other hand, the influence of MXene content on EMI shielding performance is significantly less than that of (NH_4_)_2_SO_4_, and high MXene content cannot compensate for the negative impact of low (NH_4_)_2_SO_4_ on EMI SE (Fig. 5A to G). Considering the goal of minimizing MXene filler content to reduce cost, the EMI SE of M_1.0_NS_30_PC hydrogel at the optimal MXene/(NH_4_)_2_SO_4_ ratio achieved EMI SE values of 43.1 dB in X-band, 45.8 dB in Ku-band, 50.8 dB in Ka-band, and 60.3 dB in THz-band (Fig. 5G). Notably, MNSPC DN hydrogel achieved high-performance EMI shielding across broadband (exceeding 60 dB in the THz-band) with a shallow MXene filler content of 0.12 wt %, representing the lowest conductive filler loading reported to date among flexible MXene-based hydrogel EMI shielding materials (Fig. 5H and Table S2).

The power reflection and absorption coefficients of MNSPC DN hydrogels vary depending on the MXene and (NH_4_)_2_SO_4_ contents. However, the sum of the power reflection and absorption coefficients remains nearly equal to 1, indicating that the EMI shielding mechanism of MNSPC DN hydrogels is contributed by absorption and reflection effects (Fig. 5I). The specific shielding mechanism is illustrated in Fig. 5I. First, the presence of water molecules within the hydrogel facilitates energy dissipation and enhances wave absorption, while its porous network extends the propagation pathways and promotes multiple internal reflections and absorption [11]. MXene treatment significantly enhances the conductivity of the hydrogel (Fig. S9A), forming a conductive MXene network that boosts conduction losses. Combined with polarization effects of MXene, the CS chain and the PE chain markedly enhance the EMI SE [12]. Further treatment with (NH_4_)_2_SO_4_ introduces NH_4_^+^ and SO_4_^2−^ ions, which significantly increase the conductivity of MNSPC hydrogels (Fig. S13B and C), contributing to the conduction losses [13]. Additionally, higher ion concentration intensifies the ionic polarization effect under an electric field, resulting in better absorption and scattering of electromagnetic energy within the material, achieving an approximate 20-dB enhancement in EMI SE.

**Fig. 6.**
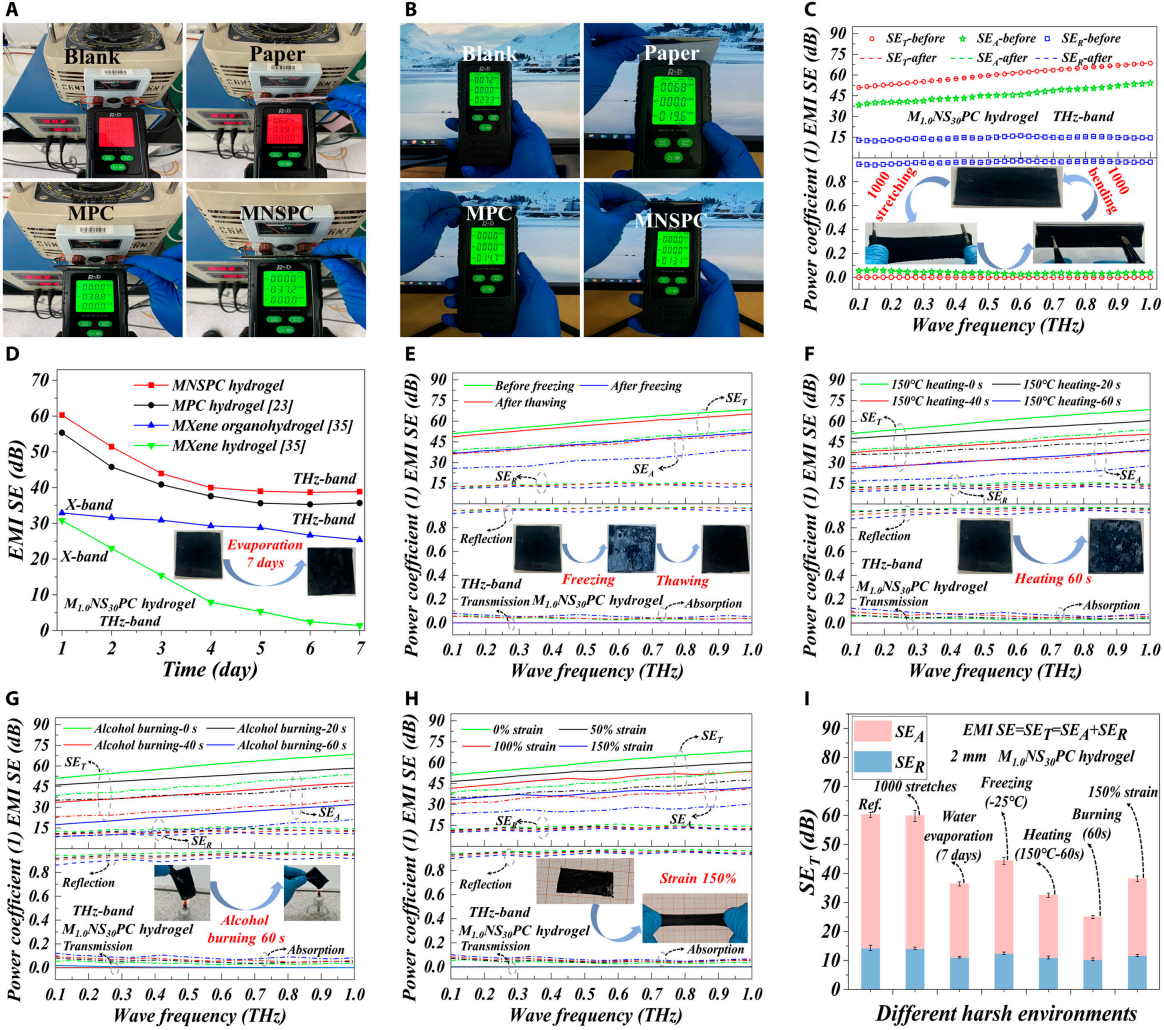
Practical application effect and environmental stability of MNSPC hydrogels in different harsh environments. Demonstrations of practical shielding effect of MNSPC hydrogel on (A) power supply and (B) computer. Effect of (C) repeated stretching and bending for 1,000 cycles, (D) water evaporation over 7 d, (E) low-temperature freezing at −30 °C over 5 h, (F) heating at 150 °C, (G) flame burning, and (H) stretching under different strain levels. (I) Comparative analysis of different environments effects on the EMI SE of MNSPC hydrogels.

The harsh working environment poses a considerable challenge to the actual SE and stability of EMI shielding materials. EMI shielding effect test results and videos involving devices such as computers and high-voltage power supplies visually demonstrate the excellent EMI shielding effect of MNSPC hydrogels (Fig. 6A and B and Movie S1). The electric field intensity, as high as 866 V/m from the high-voltage power supply (Fig. 6A) and 72 V/m from the computer (Fig. 6B), is instantaneously reduced to zero upon being shielded by MNSPC hydrogels. Benefiting from the mechanical robustness and flexibility of the MNSPC hydrogel, the EMI SE remains virtually unaffected after over 1,000 cycles of repeated stretching and bending (Fig. 6C). In practical applications, hydrogel shielding materials inevitably experience long-term water evaporation. The EMI SE of MNSPC hydrogels in the terahertz band decreased to 35.6 dB after 5 d and then remained stable (Fig. 6D). Notably, the MNSPC hydrogel demonstrated excellent stability as an organic hydrogel under water evaporation conditions and was significantly superior to traditional MXene hydrogels (Fig. 6D). In view of the extreme working conditions of certain electronic devices, such as low-temperature freezing and high-temperature heating, the EMI SE was measured before and after exposure to −30 °C freezing and 150 °C heating for various durations. After freezing at −30 °C for 5 h, the EMI SE decreased from 60.3 dB to 44.3 dB but recovered to over 60 dB upon thawing (Fig. 6E). This recovery is due to the temporary disruption of the conductive network caused by ice formation during freezing, which does not result in permanent damage (Fig. S14). A similar decrease in EMI SE was observed after heating at 150 °C, with the EMI SE dropping to 32.5 dB after 60 s of heating (Fig. 6F). High temperatures primarily lead to water evaporation, which affects the internal structure of the hydrogel. Yet, it does not reach the decomposition temperature of the MXene or (NH_4_)_2_SO_4_ components (Fig. S14). However, the reduction in EMI SE under alcohol burner flame exposure (down to 24.9 dB) was much more pronounced than at 150 °C heating environment (Fig. 6G), because the inner and outer flame temperatures of the alcohol burner exceed 500 °C, leading to the rapid evaporation of water from the hydrogel and the decrease in conductivity (Fig. S14). Moreover, the elevated temperature causes decomposition of the hydrogel matrix components, resulting in structural damage to the hydrogel system. The EMI SE results under varying strain levels demonstrated the advantages of MNSPC hydrogels in practical shielding applications. Even under a significant strain of 150%, the EMI SE in the terahertz band remained at 38.2 dB (with a shielding efficiency exceeding 99%) (Fig. 6H), surpassing that of most reported conductive EMI materials [20,24,81–86]. Moreover, this performance was achieved with an ultra-low conductive filler content of 0.12 wt % (Fig. 5H).

Clearly, under extreme conditions such as water evaporation, low-temperature freezing, flame exposure, high-temperature heating, and tensile strain, the MNSPC hydrogel exhibited varying degrees of reduction in EMI SE (Fig. 6I). Nevertheless, due to its conductivity in the range of 0.06 to 3.58 S/m (Fig. S14), it continues to demonstrate an EMI SE exceeding 20 dB under different harsh environments, which surpasses the commercial application threshold. This highlights the significant potential of MNSPC hydrogels for EMI shielding applications under harsh environmental conditions and complex structural requirements.

### Infrared stealth performance and environmental stability

**Fig. 7.**
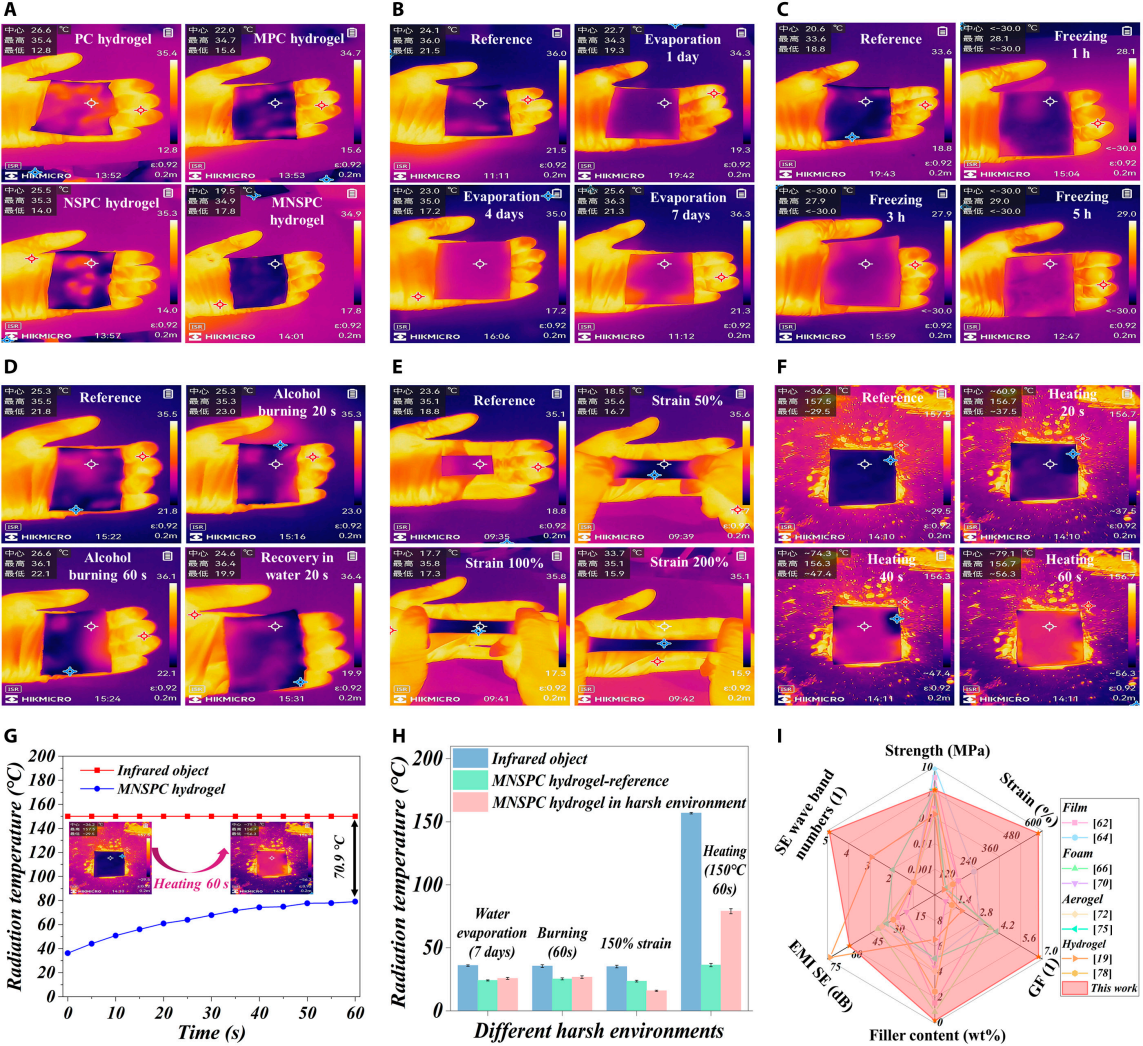
Infrared stealth performance environmental stability of MNSPC hydrogels in different harsh environments. (A) Infrared stealth performance of different hydrogel types. Effect of harsh environments such as (B) water evaporation, (C) low-temperature freezing, (D) flame burning, (E) stretching under different strain levels, and (F) high-temperature heating on the infrared stealth performance of MNSPC hydrogels. (G) Radiation temperature curves of MNSPC hydrogels on the thermal stages of 150 °C. (H) Comparison of radiation temperature of MNSPC hydrogels under different harsh environments. (I) Comparison of comprehensive performance between MNSPC hydrogel and typical EMI shielding materials.

Infrared sensors easily detect objects with temperatures above absolute zero due to continuous thermal radiation. The infrared stealth properties of MNSPC hydrogels with 2-mm thickness were evaluated using a thermal imaging camera, which is crucial for expanding their potential applications in aerospace and military fields. The radiation temperature of the hand area covered by MNSPC hydrogel decreased from 34.9 °C to 19.5 °C compared to an infrared target, demonstrating a temperature distribution similar to that of the surrounding environment, thereby providing direct evidence for excellent infrared stealth performance of the MNSPC hydrogel (Fig. 7A). Compared with PC, MPC, and NSPC hydrogels, the MNSPC hydrogel treated with the MXene/(NH₄)₂SO₄ strategy exhibited significantly improved infrared stealth, arising from the synergistic role of the MXene conductive network for electromagnetic to thermal energy conversion and the structural changes in the hydrogel induced by (NH₄)₂SO₄ (Figs. S3 and S9).

Infrared stealth materials inevitably encounter harsh working environments in practical applications, and their exceptional environmental stability is critical for expanding their application scenarios and enhancing market competitiveness. Hydrogels face the inevitable issue of prolonged water evaporation in practical applications due to the dependence on their water-rich environment. The infrared stealth performance of the MNSPC hydrogel decreased after water evaporation (the radiation temperature of covered hydrogel increased from 24.1 °C to 25.6 °C). However, even after 7 d of prolonged water evaporation, the infrared target temperature decreased from 36.3 °C to 25.6 °C, still exhibiting excellent infrared stealth (Fig. 7B). Under a low-temperature environment of −30 °C, the hydrogel underwent freezing, yet the frozen MNSPC hydrogel continued to show good infrared stealth performance, and almost unaffected by the increase in freezing time (Fig. 7C). After exposure to the alcohol lamp flame for 60 s, significant degradation in infrared stealth of MNSPC hydrogels was observed due to the water evaporation and structural degradation of the hydrogel caused by the flame temperature exceeding 500 °C. However, when rehydrated in a water-rich environment for 20 s, the infrared stealth performance was restored (Fig. 7D). Interestingly, under 150% tensile strain, a marked increase in infrared shielding performance was observed, with the radiation temperature of the MNSPC hydrogel-covered area decreasing from 23.6 °C to 16.9 °C (Fig. 7E). This phenomenon can be attributed to the enhanced infrared scattering and absorption effects caused by the increased propagation path and the increased structural nonuniformity of MNSPC hydrogels.

Furthermore, the infrared stealth and thermal insulation properties of the MNSPC hydrogel were tested under high-temperature conditions using a constant-temperature heating platform. Under a high-temperature environment of 150 °C, the temperature of MNSPC hydrogel gradually rose from 33.6 °C to 79.1 °C within 60 s (Fig. 7F). Compared to the infrared target at 150 °C, the radiation temperature of the hydrogel-covered area decreased by approximately 70.9 °C, demonstrating excellent thermal insulation performance and stability (Fig. 7G). Although infrared stealth performance showed varying degrees of change in different harsh environments, including water evaporation, low-temperature freezing, flame burning, high-strain stretching, and high-temperature heating, it still maintained excellent infrared stealth (Fig. 7H). Compared with typical shielding materials such as films, foams, aerogels, and hydrogels, the comprehensive performance of MNSPC hydrogels ranks among the best (Figs. 3K, 5H, and 7I).

## Discussion

In summary, an MNSPC DN hydrogel with strong mechanical sensing, stable high EMI shielding, and infrared stealth performance under various harsh working environments has been designed and produced using MXene/(NH₄)₂SO₄-treated strategy-assisted ultrasonic dispersion and thermal polymerization method. (NH₄)₂SO₄ triggered the Hofmeister effect and induced molecular entanglement of CS within the hydrogel, resulting in about 3-fold enhancement in mechanical properties. The MXene nanosheets were induced to align orderly the porous network structure in the hydrogel, forming a continuous MXene conductive pathway, in synergy with (NH₄)₂SO₄ to significantly enhance the conductivity. The MXene/(NH₄)₂SO₄ synergistic effect enables the MNSPC DN hydrogel to achieve excellent mechanical properties (strain with 495%, strength with 1.24 MPa, modulus with 0.49 MPa, and toughness with 3.19 MJ·m^−3^), ultra-wideband high-performance EMI shielding across the X-band, Ka-band, Ku-band, and THz-band with 60.3 dB, and exceptional infrared stealth performance, with only 0.12 wt % low conductive filler content and a thickness of 2 mm. Meanwhile, benefiting from the high conductivity, thermal stability, composite network structure, and excellent mechanical properties of MNSPC DN hydrogels, it still maintained EMI SE well above 20 dB and excellent infrared stealth performance under various harsh conditions such as repeated stretching, prolonged water evaporation, low-temperature freezing, high-temperature heating, flame exposure, and 150% tensile strain. Moreover, the easy processability of the MNSPC DN hydrogels with high GF is of great significance for scalable applications in electronic devices and soft robotics with complex shapes. In short, the MNSPC DN hydrogels with exceptional design and MXene/(NH₄)₂SO₄ strategy exhibit great application potential for scalable aerospace, military, soft robotics, and wearable electronic devices for the sake of its excellent mechanical sensing performance, stable EMI shielding and infrared stealth effects resistant to various harsh environments, low cost with 0.12 wt % conductive filler, and easy processability.

## Methods

### Materials

AA [99%, containing 200 ppm (parts per million) MEHQ as inhibitor], DMAEMA (99%, containing 1,000 ppm MEHQ as inhibitor), 2,2′-azobis(2-methyl-propionamidine) dihydrochloride (V-50,97%), and ammonium sulfate [(NH_4_)_2_SO_4_, 99%] were purchased from Aladdin Reagent Co. Ltd., China. MBAA (99%) was purchased from Sigma-Aldrich. Short-chain CS [degree of deacetylation >90%, viscosity 45 mPa·s for 1% (w/v) solution] was purchased from Jinhu Company, China. Ti_3_C_2_ MXene dispersion was obtained from 11 Technology Co. Ltd., Jilin, China.

### Fabrication of DN MNSPC hydrogel

The MNSPC hydrogels were fabricated using the following steps. Firstly, 4 g of AA, 0.5 g of DMAEMA, 0.4 g of CS, 2.5 mg of MBAA, 50 μl of 0.8 M V-50 solution, and varying volumes (0, 0.25, 0.5, and 0.75 ml) of 20 mg/ml MXene dispersion were mixed, and deionized water was added to adjust the total volume of precursor mixture to 10 ml. The mass fraction of MXene can be calculated based on the MXene content per unit volume of hydrogel:

$$\left(\mathrm{wt}\%\right)\left(\text{MXene}\right)=\frac{\rho_{M}V_{H}}{M_{H}}$$ (1)

where $\rho_{M}$ refers to the MXene content per unit volume of hydrogel, and $M_{H}$ and $V_{H}$ represent the mass and volume of hydrogel.

This precursor mixture was then sonicated in an ice bath using a SCIENTZ-IID ultrasonic homogenizer (Scientz Biotechnology Co. Ltd., Ningbo) at 240 W for 30 min to ensure uniform dispersion of the MXene. The mixture was degassed using a TMV-200T Vacuum Mixer (Smida Intelligent Equipment Co. Ltd., Shenzhen) at 1.5 kPa and 1,000 rpm for 3 min. Following this, the mixture was injected into a mold composed of 2 glass plates separated by a silicone rubber spacer to obtain hydrogel sheets. The polymerization of the PE chains was initiated at 50 °C for 12 h, with MBAA acting as the cross-linker and V-50 as the thermal initiator. Finally, the MNSPC composite hydrogels underwent salting out in an (NH_4_)_2_SO_4_ solution for 72 h to establish the CS ionic network and trigger the Hofmeister effect, resulting in the formation of the MNSPC DN hydrogels.

### Materials characterizations

SEM images of the hydrogels and EDS were captured by Gemini SEM 500 (ZEISS, Germany) at accelerating voltages of 10 and 8 kV, respectively. TEM images of the Ti_3_C_2_ MXene nanosheets were obtained using an HT7700 Exalens (HITACHI, Japan) at an accelerating voltage of 100 kV. Crystallographic data of the samples were recorded using XRD on Theta/Theta Rotating anode X-ray Diffractometer (TTR III, Japan). Raman spectroscopy measurements were performed using the Confocal Raman System-Horiba LabRAM SoLeil (Horiba, Japan) equipped with a 785-nm laser, and the spectra were recorded in the range of 1,500 to 100 cm^−1^. FTIR of the samples was performed in the range of 4,000 to 500 cm^−1^ on a Nicolet 8700 IR spectrophotometer (Thermo Scientific, USA). XPS measurements were performed on X-ray Photoelectron Spectrometer-Kratos AXIS SUPRA^+^ (Shimadzu, Japan).

### Mechanical test

Mechanical properties were measured by an HY-0350 testing machine (HengYi Instrument Co. Ltd., Shanghai). The samples were stamped into a dumbbell shape with 2-mm inner width, 20-mm gauge length, and 1- to 2-mm thickness for the test. To reduce the effect of dehydration, the tensile rate was set to 50 mm/s. All mechanical properties were tested on no less than 3 samples, average values were selected as the final results, and standard deviations were marked as error bars in the figures.

### Strain-sensing test

The samples were cut into a rectangular shape with a width of 6 mm, a thickness of 1 to 2 mm, and a gauge length of 30 mm for the strain-sensing test. The GF was used to quantify the strain sensitivity of the hydrogels. The resistance change of the samples at different strains was measured using a TH2832 LCR meter, while the samples were loaded and unloaded by the finger, wrist, and balloon. The relative changes in stress and resistance determined GF:

$$\mathrm{GF}=\frac{R-R_{0}}{R_{0}}\frac{1}{\varepsilon -\varepsilon_{0}}$$ (2)

where $R_{0}$ and R represent the initial resistance and real-time resistance of the sample, respectively, and $\varepsilon_{0}$ and ε represent the initial strain and real-time strain of the sample, respectively.

To further demonstrate the functional capability of the hydrogels as sensors, the gels were integrated into a circuit with an LED. Theoretically, stretching the gel would increase its resistance, causing a decrease in the current flowing through the LED, subsequently reducing its brightness.

### Electromagnetic shielding test in different EM frequency bands

Electromagnetic shielding bands of hydrogels in this work mainly focus on the X-band (8.2 to 12.4 GHz), Ku-band (12.4 to 18 GHz), Ka-band (90.5 to 40 GHz), and THz-band (0.1 to 1.0 THz). EMI SE values of hydrogels in X-band, Ku-band, and Ka-band were measured by microwave test system (Anritsu-MS46322B vector network analyzer with a range of 1 MHz to 43.5 GHz), and the corresponding samples were cut into a rectangular shape with 50 mm × 50 mm (length × width) for measurements. The power transmission $T_{p}$, reflection $R_{p}$, and absorption $A_{p}$ coefficients in the hydrogels were calculated by *S* parameters (forward $S_{21}$ and reverse $S_{12}$ transmission coefficients, input $S_{22}$ and output $S_{11}$ reflection coefficients) based on Eqs. (3) to (5), and corresponding EMI SE values of hydrogels were obtained by power transmission and reflection coefficients based on Eqs. (6) to (8).

$$T_{p}={\left|S_{12}\right|}^{2}={\left|S_{21}\right|}^{2}$$ (3)

$$R_{p}={\left|S_{11}\right|}^{2}={\left|S_{22}\right|}^{2}$$ (4)

$$A_{p}=1-T_{p}-R_{p}$$ (5)

$$\mathrm{EMI}\;\mathrm{SE}={\mathrm{SE}}_{T}=-10\log\left(T_{p}\right)$$ (6)

$${\mathrm{SE}}_{R}=-10\log\left(\frac{1}{1-R_{p}}\right)$$ (7)

$${\mathrm{SE}}_{A}={\mathrm{SE}}_{T}-{\mathrm{SE}}_{R}$$ (8)

where ${\mathrm{SE}}_{T}$, ${\mathrm{SE}}_{R}$, and ${\mathrm{SE}}_{A}$ represent the overall SE, reflection, and absorption effectiveness (in dB), respectively.

EMI SE values of hydrogels in THz-band were measured using the terahertz time domain spectroscopy (THz-TDS) test system (TAS7400TS). Considering the non-negligible influence of water vapor on terahertz waves, the test environment was controlled below 5% relative humidity (RH) by an air dryer equipped with a temperature and humidity recording transmitter (G95-4PS, Hangzhou Loggertech Co. Ltd.). Overall shielding, reflection, absorption effectiveness, and EMI SE of MPC hydrogels were measured and analyzed in the transmission mode and reflection mode of THz-TDS system based on Eqs. (9) and (10).

$$\mathrm{EMI}\;\mathrm{SE}={\mathrm{SE}}_{T}=-20\log\left(\frac{E_{s}}{E_{a}}\right)$$ (9)

$${\mathrm{SE}}_{R}=-10\log\left(\frac{E_{r}}{E_{i}}\right)$$ (10)

where $E_{s}$ and $E_{a}$ refer to the terahertz pulse intensity through MPC hydrogel and air in the transmitted light path, respectively, and $E_{r}$ and $E_{i}$ represent the incident and reflected terahertz pulse intensity of hydrogels in reflected optical path, respectively.

### Infrared stealth performance test

Infrared stealth performance of hydrogels in this work was tested via infrared thermal camera (H21PROS+, China), with the infrared wavelength range spanning from 8 to 14 μm. The corresponding samples were cut into a rectangular shape with 50 mm × 50 mm (length × width) for measurements. Heating tests in the infrared range were conducted using a stainless steel digital constant-temperature heating plate (LC-DB-AB, China), which generated a 150 °C high-temperature environment while minimizing the influence of surface materials on the infrared measurements.

### Statistical analysis

All data were presented as the means ± standard deviation.

## Data Availability

Additional data are available from the corresponding author upon request. Data supporting the findings of this study are available within the article and its Supplementary Materials.
