# Supplementary material for: Multifunctional Hydrogels with Broadband Electromagnetic Interference Shielding and Infrared Stealth Performance in Harsh Environments with Low Conductive Filler Content
Source: Research (Wash D C). 2026 Feb 6;9:1020. doi: 10.34133/research.1020 (PMC12877210; doi:10.34133/research.1020)
Supplement: Supplementary 1 — Figs. S1 to S14 Tables S1 and S2 Movie S1 [file research.1020.f1.zip › Revised Supplemental Information (1).docx]

**Supporting Information for**

**Multifunctional Hydrogels with Broadband Electromagnetic Interference Shielding and Infrared Stealth Performance in Harsh Environments with Low Conductive Filler Content**

Wenchong Ouyang^1,3a^, Lin Mei^2,5a^, Limin Xu^6^, Chengwei Zhao^7^, Yu Bai^8^, Ziyang Zhao^3^, Rongxin Tang^1,3*^, Tianzhi Luo^5*^, Zhengwei Wu^4*^

^1^School of Information Engineering, Nanchang University, Nanchang, Jiangxi 330031, China.

^2^School of Nuclear Science and Technology, University of Science and Technology of China, Hefei 230026, China.

^3^Institute of Space Science and Technology, Nanchang University, Nanchang, Jiangxi 330031, China

^4^Joint Laboratory of Plasma Application Technology, Institute of Advanced Technology, University of Science and Technology of China, Hefei 230031, China.

^5^CAS Key Laboratory of Mechanical Behavior and Design of Materials, Department of Modern Mechanics, CAS Center for Excellence in Complex System Mechanics, University of Science and Technology of China, Hefei 230026, China.

^6^Advanced Institute of Photonics Technology, School of Information Engineering, Guangdong University of Technology, Guangzhou 510006, China.

^7^Key Laboratory of Information and Structure Efficiency in Extreme Environment, the Ministry of Education of China, Xidian University, Xi’an 710071, China.

^8^Experimental Center of Engineering and Materials Sciences, University of Science and Technology of China, Hefei 230026, China.

^a^These authors contributed equally: Wenchong Ouyang, Lin Mei.

*Correspondence to: Rongxin Tang (rongxint@ncu.edu.cn), Tianzhi Luo (tzluo@ustc.edu.cn), Zhengwei Wu (wuzw@ustc.edu.cn).

**1.1 Figures**


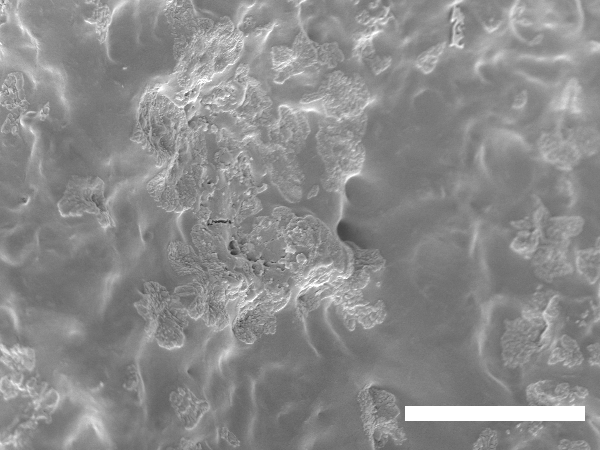


**Figure S1.** SEM image of MNSPC hydrogel (0.5 mg/mL MXene and 43% (NH_4_)_2_SO_4_) without discharging (NH_4_)_2_SO_4_ (scare bar: 10 μm).


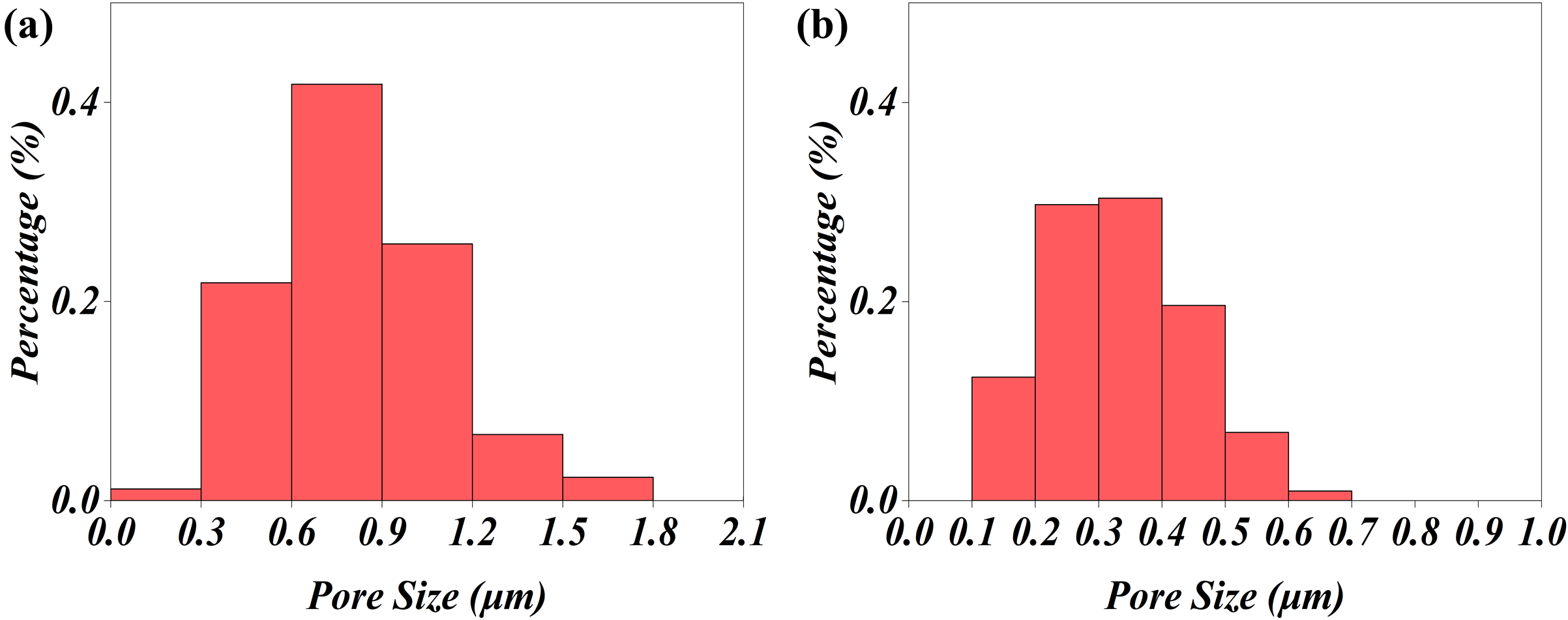


**Figure S2.** Pore size of (a) MPC (0.5 mg/mL MXene and 0% (NH_4_)_2_SO_4_) and (b) MNSPC (0.5 mg/mL MXene and 43% (NH_4_)_2_SO_4_) hydrogels.


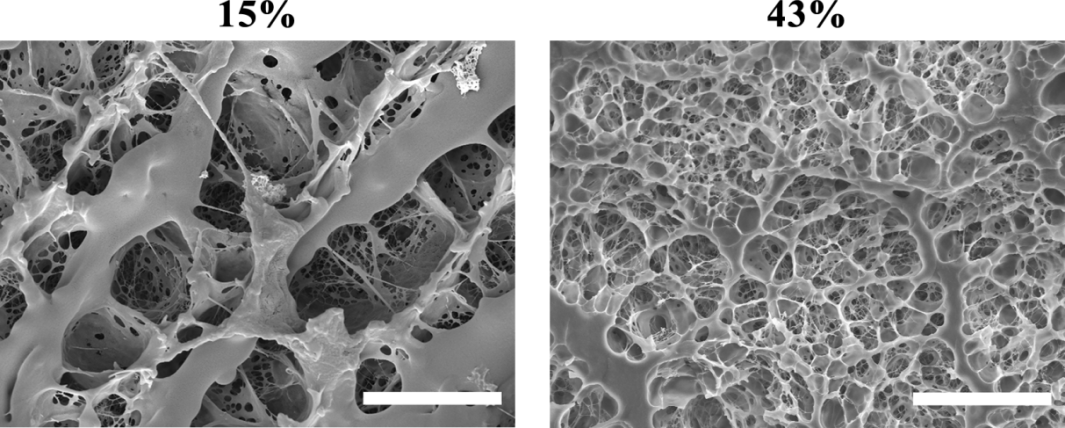


**Figure S3.** SEM image of MNSPC hydrogels (0.5 mg/mL MXene) with (a) 15% and (b) 43% (NH_4_)_2_SO_4_ (scare bar: 5 μm).


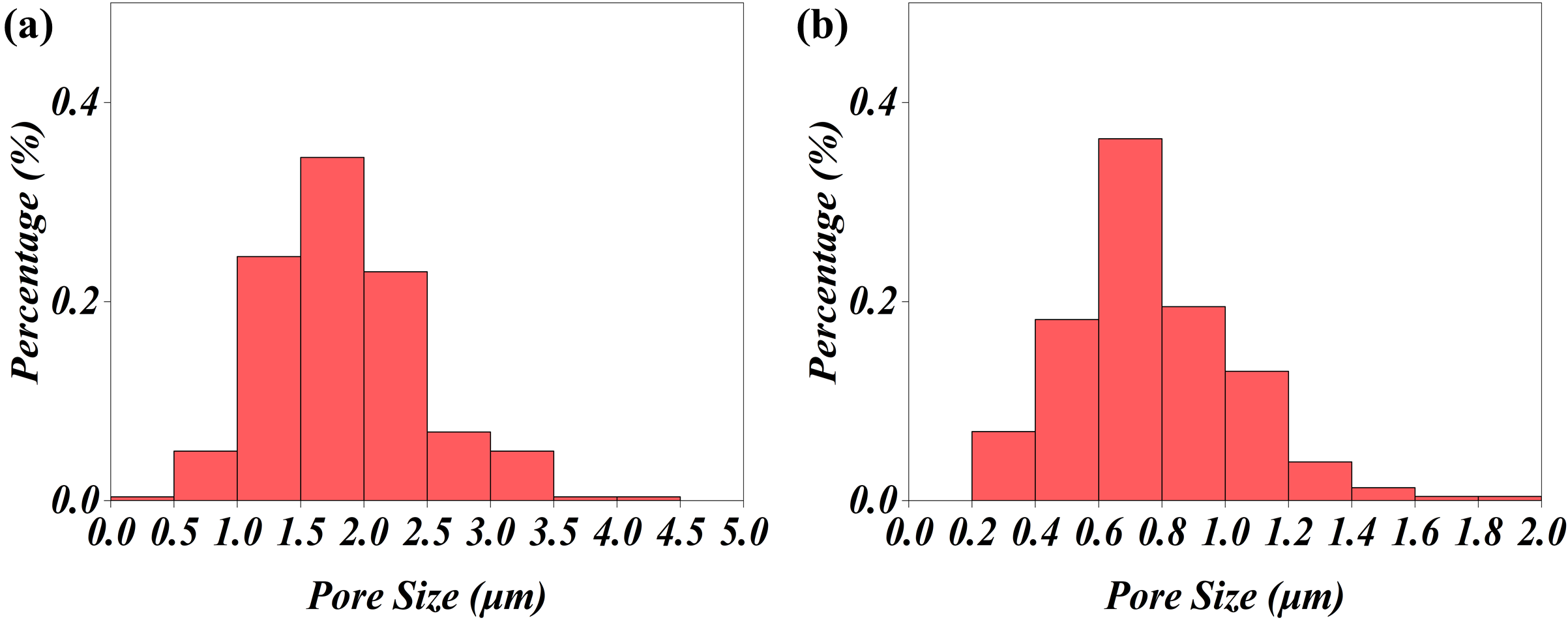


**Figure S4.** Pore size of MNSPC hydrogels with different (NH_4_)_2_SO_4_ concentrations. (a) 15%, (b) 43%.


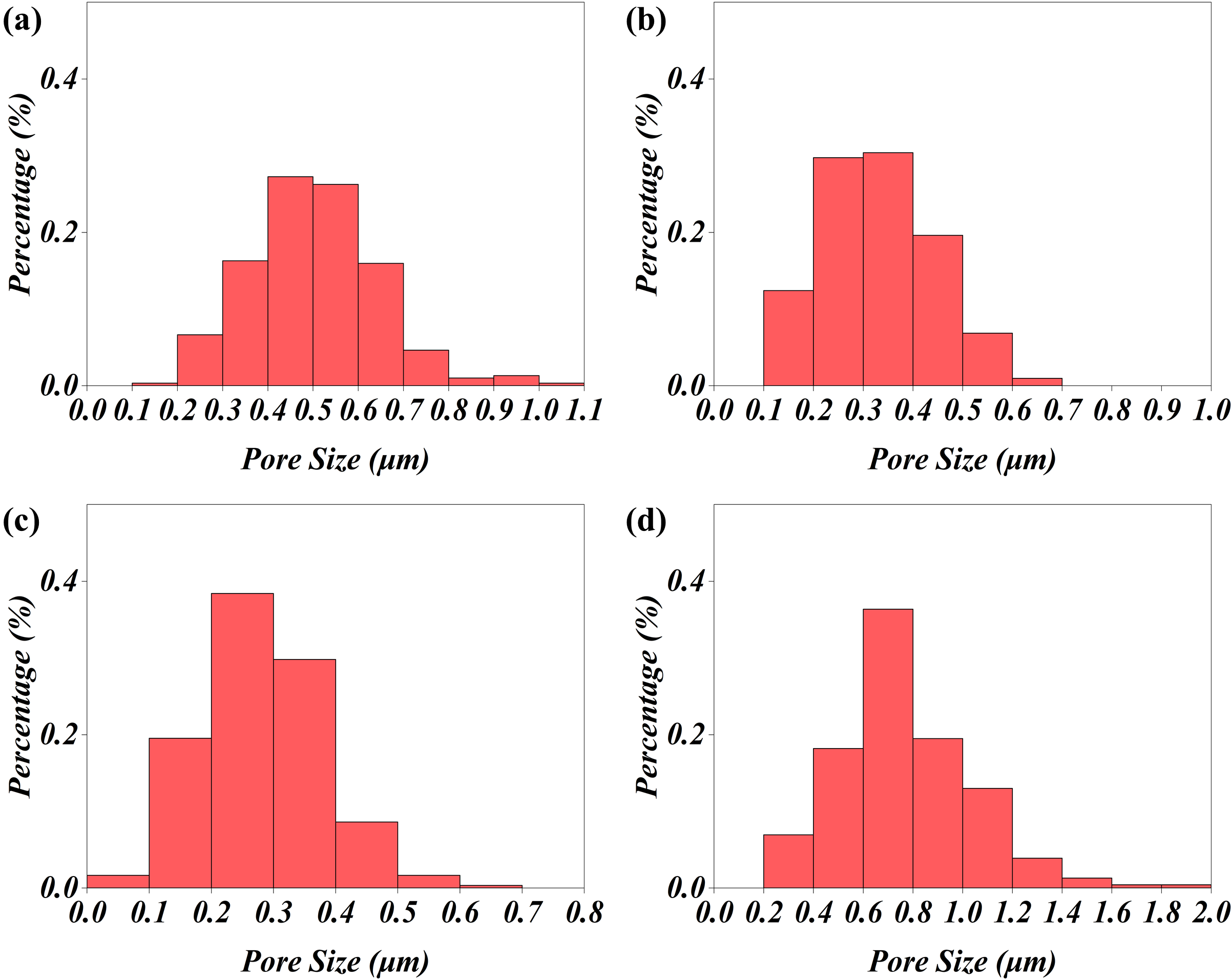


**Figure S5.** Pore size of MNSPC hydrogels (43% (NH_4_)_2_SO_4_) with different MXene concentrations. (a) 0.0 mg/mL, (b) 0.5 mg/mL, (c) 1.0 mg/mL, (d) 1.5 mg/mL.


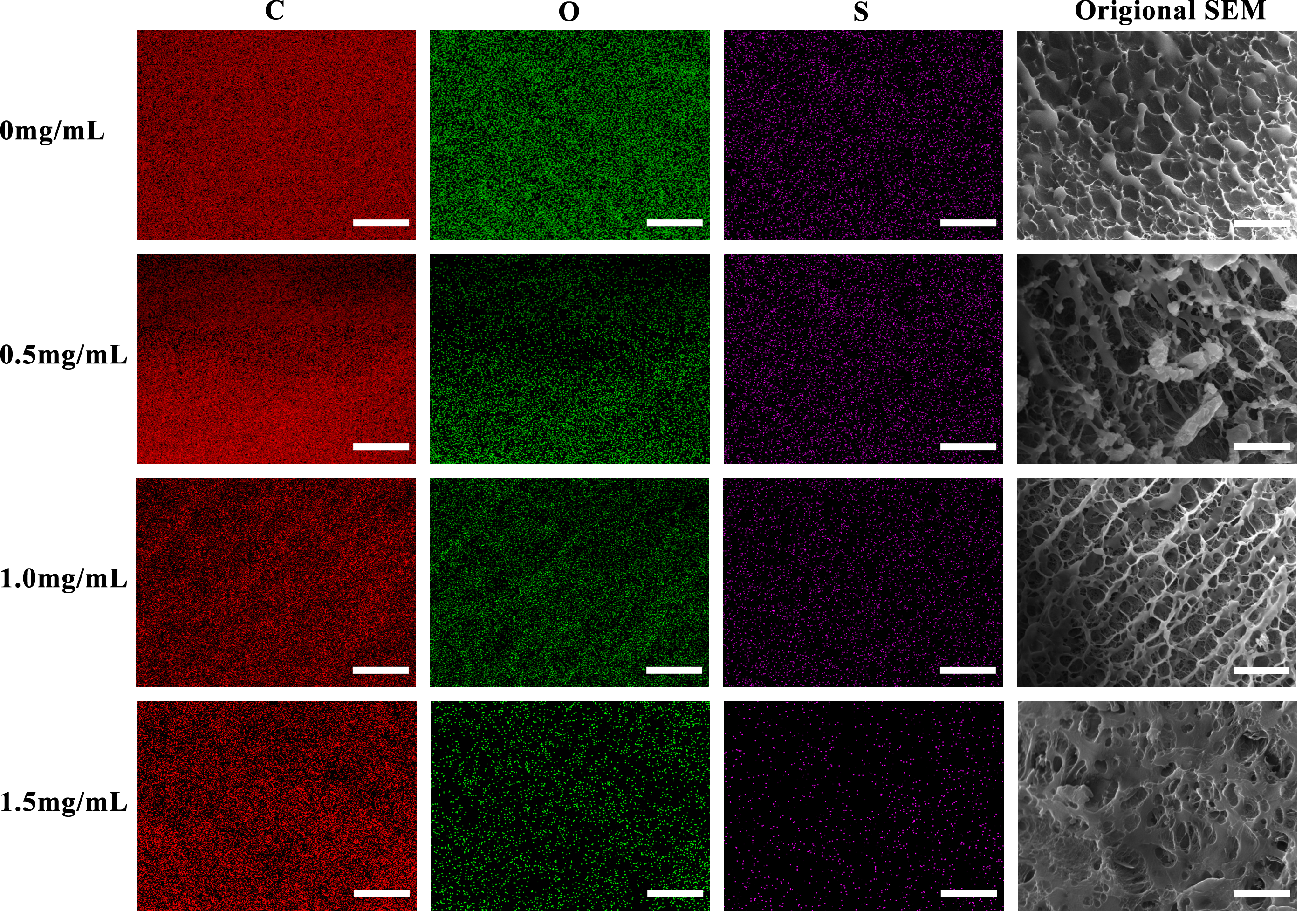


**Figure S6.** EDS mapping of C, O and S elements and the original SEM figure of MNSPC hydrogels (43% (NH_4_)_2_SO_4_) with different MXene contents after salting out (scare bar: 10 μm).


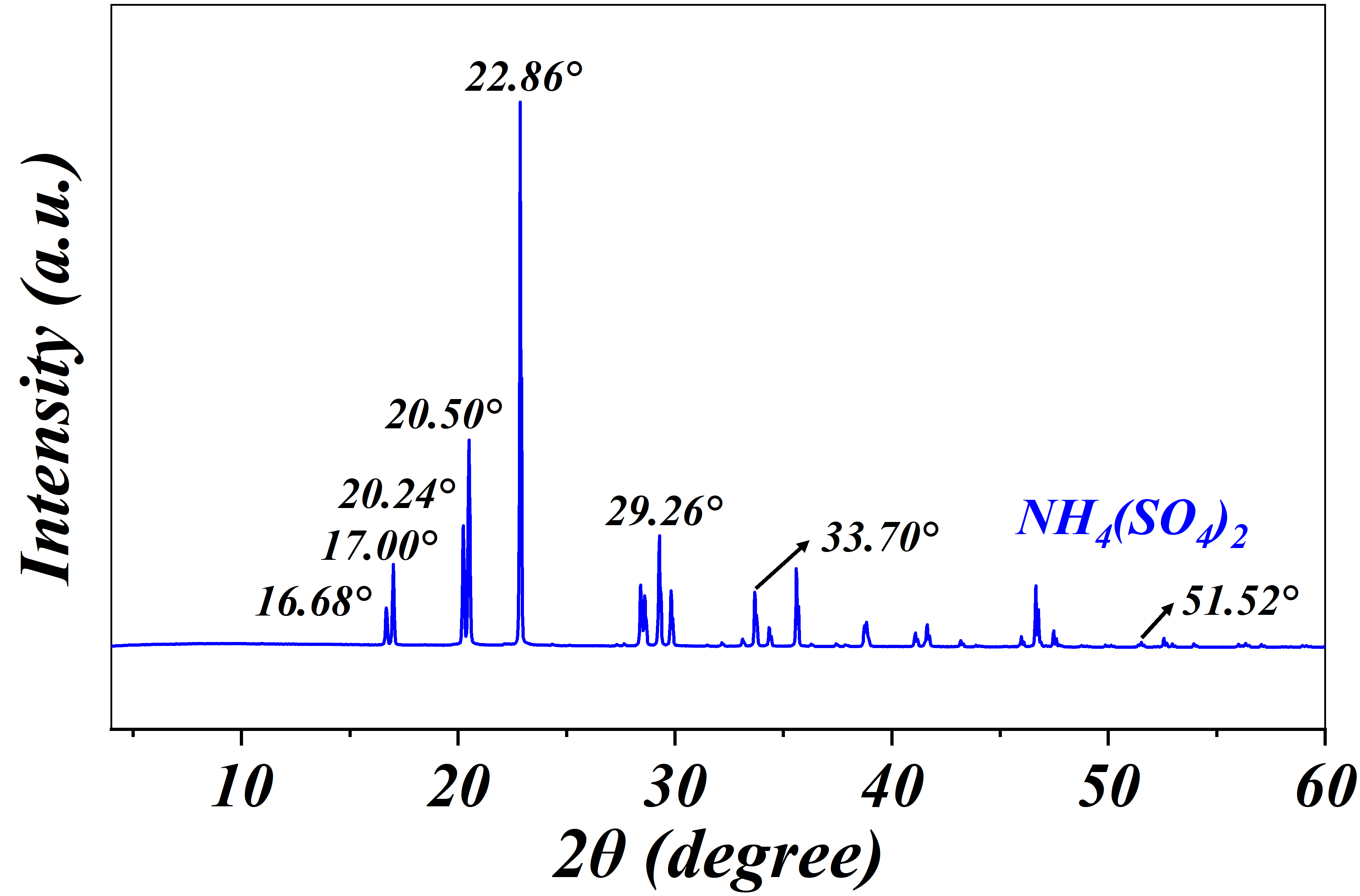


**Figure S7.** XRD characterization results of (NH_4_)_2_SO_4_.


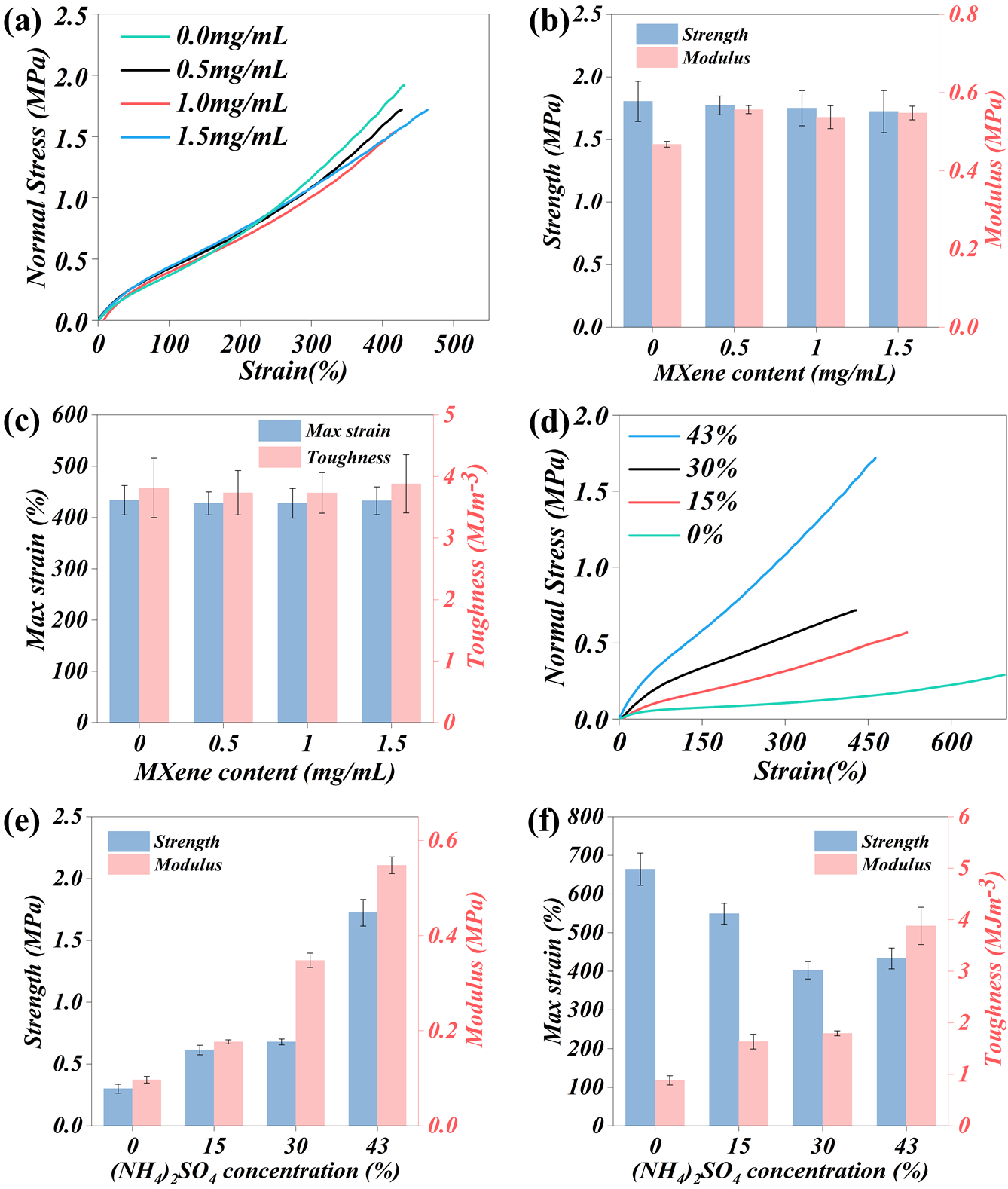


**Figure S8.** Influence of MXene (a-c) and (NH_4_)_2_SO_4_ (d-f) contents on the comprehensive mechanical properties of MNSPC hydrogels. (a) Stress-strain curves, (b) tensile strength and modulus, (c) tensile strain and toughness of MNSPC hydrogels under different MXene contens ((NH_4_)_2_SO_4_: 43%), (d) stress-strain curves, (e) tensile strength and modulus, (f) tensile strain and toughness of MNSPC hydrogels under different (NH_4_)_2_SO_4_ contens (MXene: 1.5 mg/mL).


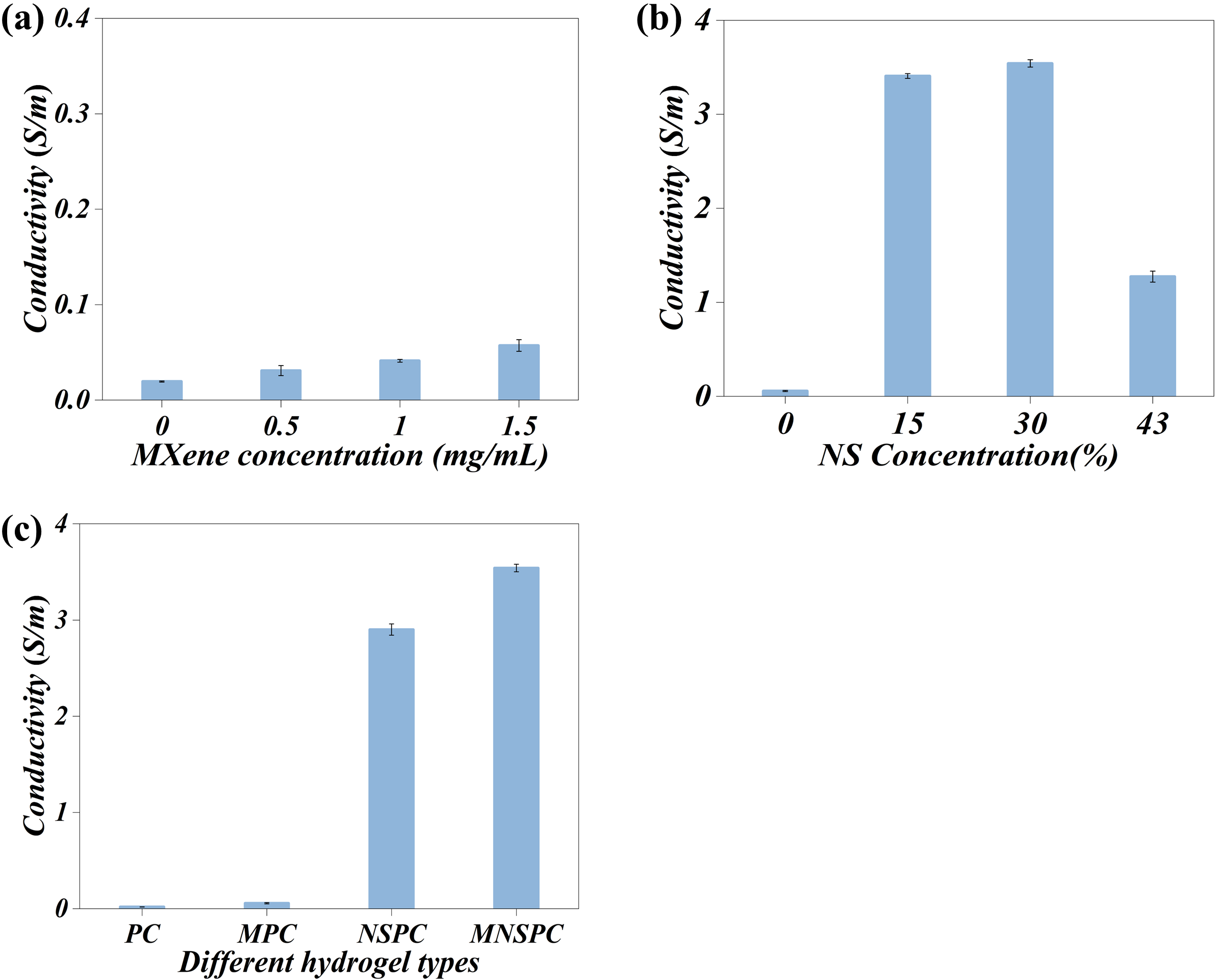


**Figure S9.** Conductivity of hydrogels under different (a) MXene concentrations (MPC hydrogels), (b) (NH_4_)_2_SO_4_ concentrations (MNSPC hydrogels), and (c) hydrogel types.


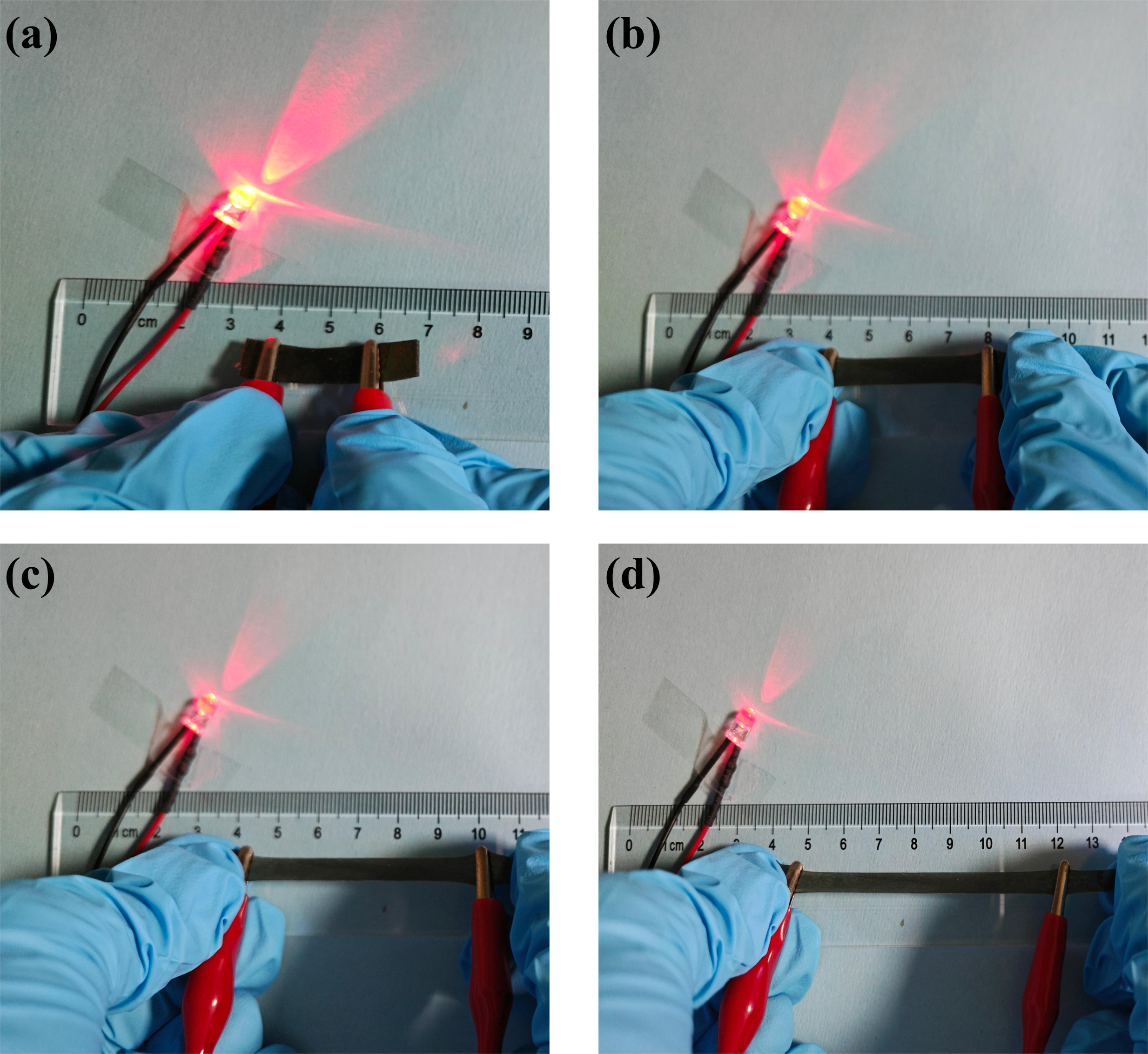


**Figure S10.** Brightness changes of light-emitting diodes under different strains.


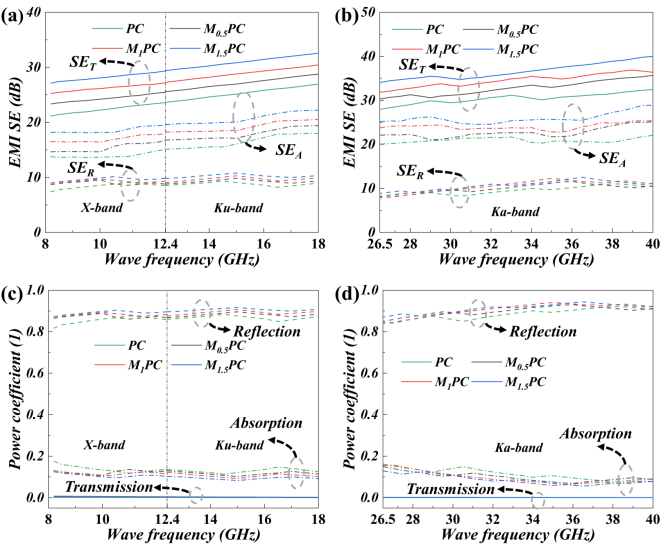


**Figure S11.** EMI SE and power coefficient of hydrogels in different wavebands under different MXene contents. EMI SE of hydrogels in (a) X-band, Ku-band, and (b) Ka-band, power coefficient of hydrogels in (c) X-band, Ku-band, and (d) Ka-band.


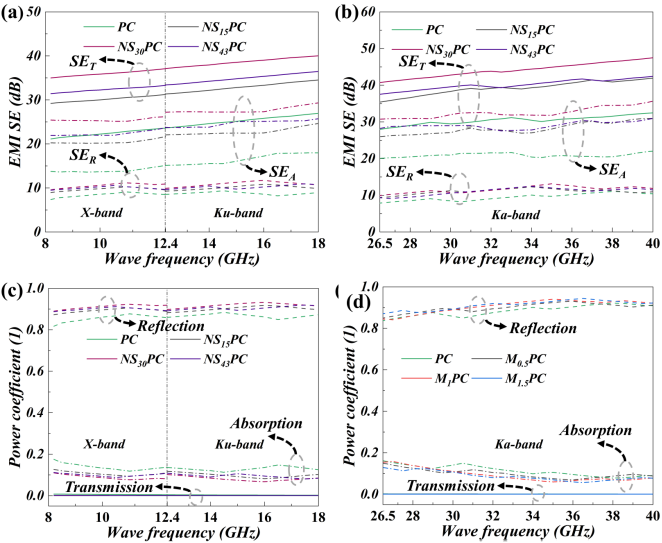


**Figure S12.** EMI SE and power coefficient of hydrogels in different wavebands under different (NH_4_)_2_SO_4_ contents. EMI SE of hydrogels in (a) X-band, Ku-band, and (b) Ka-band, power coefficient of hydrogels in (c) X-band, Ku-band, and (d) Ka-band.


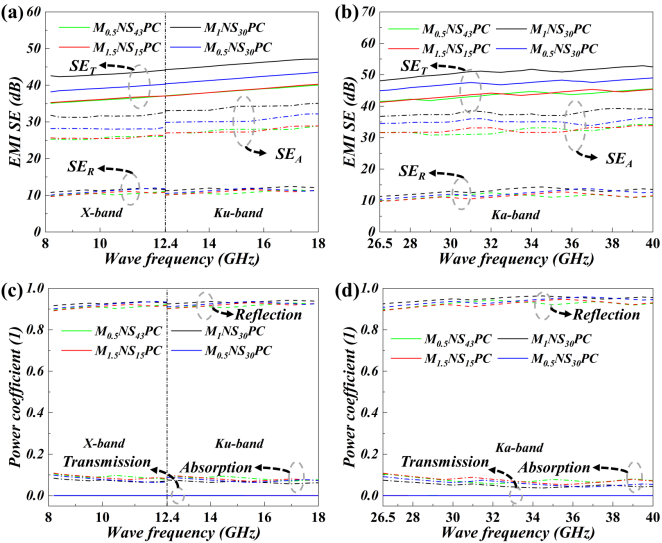


**Figure S13.** EMI SE and power coefficient of MNSPC hydrogels in different wavebands under different MXene/(NH_4_)_2_SO_4_ ratios. EMI SE of hydrogels in (a) X-band, Ku-band, and (b) Ka-band, power coefficient of hydrogels in (c) X-band, Ku-band, and (d) Ka-band.

**
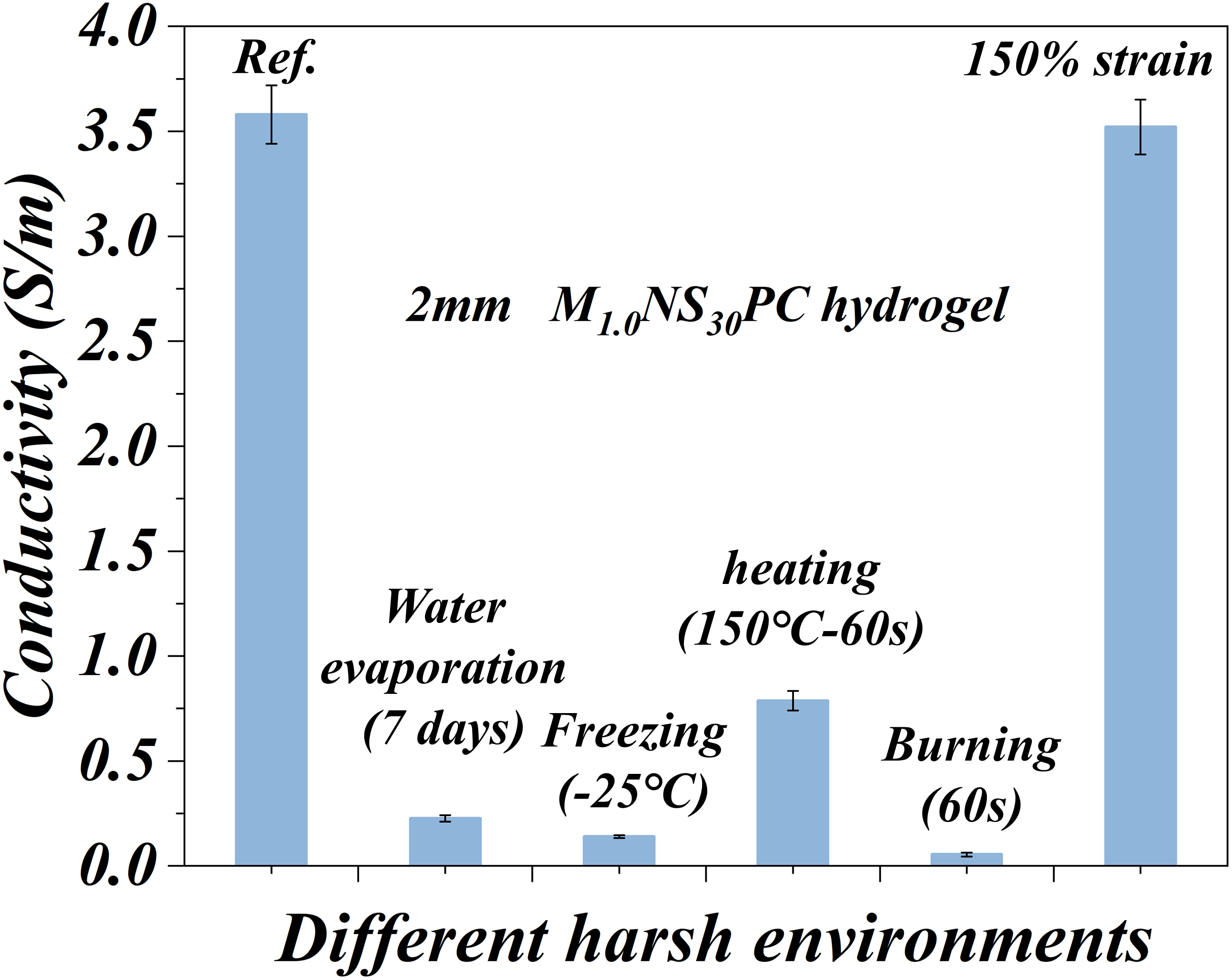
**

**Figure S14.** Comparison of conductivity of MNSPC hydrogels under different harsh environments.

**1.2 Tables**

**Table S1.** Comparison of comprehensive mechanical properties of MNSPC hydrogels with other typical hydrogel electromagnetic shielding materials.

| **Hydrogel types** | **Filler content**  **(wt%)** | **Strength**  **(MPa)** | **Strain**  **(%)** | **Ref.** |
| --- | --- | --- | --- | --- |
|  |  |  |  |  |
| CNT@LM/polyacrylamide/gelatin | 0.5 | 0.46 | 500 | [S1] |
| Polyacrylamide-AA | 10 | 0.095 | 40 | [S2] |
| MXene-PAA-ACC | 8.5 | 0.02 | 600 | [S3] |
| Polyacrylamide-AA | 1 | 0.12 | 6 | [S4] |
| AgNWs-MS/PVA | 20 | 0.14 | 50 | [S5] |
| Poly(PT)/PVA/p-MXene | 0.31 | 0.15 | 597 | [S6] |
| MXene/PE-CS | 0.48 | 0.47 | 747 | [S7] |
| MXene/H_2_SO_4_/PEDOT:PSS/PVA | 0.6 | 0.18 | 900 | [S8] |
| AgNWs/PAA | / | 0.15 | 1045 | [S9] |
| MXene-based organohydrogels | 1 | 0.77 | 482 | [S10] |
| MXene/glycerol/PVA | 1.17 | 1.5 | 440 | [S11] |
| MXene/PEDPT:PSS-SSD. | 1.8 | 0.2 | 78 | [S12] |
| PPy-NTs/ PVA-PEG | 1.2 | 2.12 | 101 | [S13] |
| PAM/PVA/LiCl | 1.7 | 0.08 | 380 | [S14] |
| PVA/PEG+PNT@Fe3O4/HGM@Ag | / | 0.21 | 127 | [S15] |
| PAM/HACC/PPy | / | 0.92 | 510 | [S16] |
| SA/PAM/IL@PGO | / | 0.04 | 184 | [S17] |
| MCNTs/PAA | 3 | 0.07 | 83 | [S18] |
| PG/MXene@CNF/CaCl_2_ | / | 0.23 | 831 | [S19] |
| 3D CNT/PEDOT:PSS | 0.2 | 0.05 | 10 | [S20] |

**Table S2.** Comparison of EMI SE in different waveband and corresponding conducive filler content, strength, strain and GF of our MNSPC DN hydrogels with other landmarking conductor-based shields reported in literature.

| **Materials** | **Filler content**  **(wt%)** | **Strength**  **(MPa)** | **Strain**  **(%)** | **GF**  **(1)** | **EMI SE (dB)** | | | | **Ref.** |
| --- | --- | --- | --- | --- | --- | --- | --- | --- | --- |
|  |  |  |  |  | **X** | **Ku** | **Ka** | **THz** |  |
| **Film** | | | | | | | | | |
| PCC/MXene/  PVA | / | 30 | 20 | / | 43.1 | / | / | / | [S21] |
| CNTs/rubber | 44 | 22.2 | 16 | / | 50 | / | / | / | [S22] |
| Graphene/  PDMS | 1.5 | 3.7 | 131.7 | / | 26.5 | / | / | / | [S23] |
| Cu Mesh | / | / | / | 6 | 40.4 | / | / | / | [S24] |
| MXene/PA-40 | 40 | 120 | 2.3 | / | 38.9 | / | / | 52.7 | [S25] |
| PEDOT:  PSS/PU | 5 | 9.0 | 217.4 | / | 38.6 | / | / | / | [S26] |
| **Foam** | | | | | | | | | |
| CNT/PU foam | 12 | 0.13 | 170 | / | 32 | / | / | / | [S27] |
| C-MXene@PI | 0.8 | / | / | 3.6 | 40.2 | / | / | / | [S28] |
| CNTs foam | 5 | 10.6 | 529 | / | 43.7 |  |  |  | [S29] |
| PS/CNT | 1 | 2.2 | 61.9 | / | 20.1 | / | / | / | [S30] |
| Co_3_O_4_/CNT/  Carbon | 3 | 0.023 | 80 | / | 25.6 | / | / | / | [S31] |
| CNTs/PU | 4.3 | 0.25 | 131.3 | 0.7 | 20 | / | / | / | [S32] |
| **Aerogel** | | | | | | | | | |
| ASC/rGO | 5 | 1.23 | 5 | / | 44 | / | / | / | [S33] |
| CA1-M_4_ | 4 | 0.17 | 80 | 1.2 | 37.2 | / | / | / | [S34] |
| CZn-2 | / | 0.012 | 60 | 4.0 | 46.5 | / | / | / | [S35] |
| MXene /CNFs | 26.5 | 4.9 | 3.1 | / | 54 | / | / | / | [S36] |
| C100GA-300 | / | / | / | / | 35 | / | / | 34 | [S37] |
| graphene@SiC | 30 | / | / | / | / | 47.3 | / | / | [S38] |
| CNF/SBC | / | / | / | / | / | / | / | 65 | [S39] |
| **Hydrogel** | | | | | | | | | |
| MXene- PEDOT: PSS | 3.4 | 0.12 | 5.5 | 0.4 | 51.7 | / | / | / | [S4] |
| AgNWs-MS/PVA | / | 0.14 | 50 | 1.74 | 45 | 55 | 75 | / | [S5] |
| Py-NTs/ PVA/ PEG/GA | 20 | 2.28 | 100.9 | / | 21 | / | / | / | [S40] |
| rGO/GEL | 2.4 | 1.2 | 100.4 | 1.1 | 29.4 | / | / | / | [S41] |
| PDMS@TC  /MXene | 0.42 | 15 | 3.38 | 0.45 | 49.4 | / | / | / | [S42] |
| MXene-PAA  -ACC | 10 | 0.02 | 600 | 5.2 | / | / | / | 45.3 | [S3] |
| MXene organohydrogel | 0.4 | / | / | / | 32.8 | / | / | / | [S43] |
| PAM/SA/C-  MXene | 6.5 | 3.42 | 230 | / | 26.8 | / | / | / | [S44] |
| **M_0.5_NS_30_PC DN hydrogel** | **0.06** | **1.12** | **548** | **6.87** | **39.3** | **42** | **47.2** | **57.4** | **This**  **work** |
| **M_1.0_NS_30_PC DN hydrogel** | **0.12** | **1.24** | **495** | **6.74** | **43.1** | **45.8** | **50.8** | **60.3** |  |
